# The role of influenza in the epidemiology of pneumonia

**DOI:** 10.1038/srep15314

**Published:** 2015-10-21

**Authors:** Sourya Shrestha, Betsy Foxman, Joshua Berus, Willem G. van Panhuis, Claudia Steiner, Cécile Viboud, Pejman Rohani

**Affiliations:** 1 Department of Ecology & Evolutionary Biology, University of Michigan, Ann Arbor, MI 48109, USA; 2 Center for the Study of Complex Systems, University of Michigan, Ann Arbor, MI 48109, USA; 3 Department of Epidemiology, Johns Hopkins School of Public Health, Baltimore, MD 21205, USA; 4 Department of Epidemiology, University of Michigan, Ann Arbor, MI 48109, USA; 5 Undergraduate Research Opportunity Program, University of Michigan, Ann Arbor, MI 48109, USA; 6 Department of Epidemiology, University of Pittsburgh Graduate School of Public Health, Pittsburgh PA 15261, USA; 7 Healthcare Cost and Utilization Project, Center for Delivery, Organization and Markets, Agency for Healthcare Research and Quality, U.S. Department of Health and Human Services, Rockville, MD 20850, USA; 8 Division of International Epidemiology and Population Studies, National Institutes of Health, Bethesda, MD 20892, USA; 9 Odum School of Ecology, University of Georgia, Athens, GA 30602, USA; 10 Department of Infectious Diseases, School of Veterinary Medicine, University of Georgia, Athens, GA 30602, USA; 11 Fogarty International Center, National Institutes of Health, Bethesda, MD 20892, USA

## Abstract

Interactions arising from sequential viral and bacterial infections play important roles in the epidemiological outcome of many respiratory pathogens. Influenza virus has been implicated in the pathogenesis of several respiratory bacterial pathogens commonly associated with pneumonia. Though clinical evidence supporting this interaction is unambiguous, its population-level effects—magnitude, epidemiological impact and variation during pandemic and seasonal outbreaks—remain unclear. To address these unknowns, we used longitudinal influenza and pneumonia incidence data, at different spatial resolutions and across different epidemiological periods, to infer the nature, timing and the intensity of influenza-pneumonia interaction. We used a mechanistic transmission model within a likelihood-based inference framework to carry out formal hypothesis testing. Irrespective of the source of data examined, we found that influenza infection increases the risk of pneumonia by ~100-fold. We found no support for enhanced transmission or severity impact of the interaction. For model-validation, we challenged our fitted model to make out-of-sample pneumonia predictions during pandemic and non-pandemic periods. The consistency in our inference tests carried out on several distinct datasets, and the predictive skill of our model increase confidence in our overall conclusion that influenza infection substantially enhances the risk of pneumonia, though only for a short period.

Lower respiratory infections are the leading infectious cause of human mortality, resulting in 3.2 million deaths worldwide in 2011^1^,^2^. While the etiology of such infections involves a multitude of respiratory bacteria and viruses, it is becoming increasingly evident that interactions between them play an important role^3^,^4^. Many of the bacteria associated with pneumonia^5^ can interact with respiratory viruses^6^,^7^, particularly the influenza virus^8^,^9^. A growing body of literature on challenge experiments in animal models, and *in vitro* studies have shown that influenza virus shapes the outcome of infection with many respiratory bacteria, including *Streptococcus pneumoniae*^10^,^11^,^12^,^13^,^14^,^15^, *Haemophilus influenzae*^16^,^17^, *Staphylococcus aureus*^18^,^19^,^20^,^21^, and *Klebsiella pneumoniae*^22^,^23^ indicating that a viral infection can enhance both susceptibility to and the severity of subsequent bacterial infection^8^,^24^,^25^.

Understanding the role that influenza plays on the population-level epidemiology of bacterial pneumonia remains a challenge. In particular, it is important to dissect the nature of any interaction: Is the impact of influenza infection limited to the clinical manifestation of subsequent pneumonia (severity of disease), or does it also influence its epidemiology, perhaps by increasing either host susceptibility or transmission? Additionally, the magnitude of the interaction also requires quantification. How much of the observed pneumonia incidence can be attributed to the interaction with influenza? How sensitive is this effect to influenza virus subtype or the distribution of circulating bacteria? The resolution of these issues will permit the accurate evaluation of alternative public health interventions, such as the deployment of influenza and pneumococcal vaccines, or the use of antivirals and antibiotics.

A puzzling aspect of the interaction between influenza and bacterial pneumonia, as observed in population settings, is the variability in its signal. Histological examinations and autopsy reports of the 1918 influenza pandemic victims shows evidence of bacterial invasion in more than 90% of cases^26^, and, taken together with the accounts of bacterial pneumonia cases in army camps across the USA during the 1918 pandemic^27^, suggest high rates of bacterial co-infection in influenza patients, indicating an extremely strong interaction. Similarly, hospital admissions during the 1957 Asian^28^,^29^, the 1968 Hong Kong^30^,^31^, and the 2009^32^ influenza pandemics also indicate a moderate to strong association. In contrast, studies that have tested for an association during non-pandemic periods have concluded the interaction is modest or non-existent^33^,^34^,^35^.

Such variability in the footprints of an interaction can arise from two fundamentally different mechanisms. First, the intensity of interaction between influenza and bacterial pneumonia may itself vary widely owing to the virulence and severity of the circulating influenza strains^36^, variability in the bacterial virulence factors^37^, or possibly the difference in the intensity of interaction between the specific virus-bacterium pairing^7^. Alternatively, the variability in the signature may be a reflection of the magnitude of influenza epidemics. The variability in influenza epidemics can depend on the characteristics of the dominant viral strain and population immunity. Thus, the dynamical consequences of an influenza-driven interaction can be more discernible when the influenza epidemics are larger, even when the intensity of the interaction is not necessarily different^38^.

In a previous paper, we established the epidemiological consequences of influenza infection on pneumococcal pneumonia in a contemporary population in the US^38^. Here, we broadened our scope to understand the association between influenza and bacterial pneumonia more generally. Additionally, we turned our attention to three distinct sets of incidence data that differ in the identity of the circulating influenza viral subtype, as well as in influenza epidemic size. Dataset 1 consisted of influenza and pneumonia weekly incidence from the State of Illinois before (dataset 1A: 1990–1997), and following (dataset 1B: 2000–2009) the roll-out of the pneumococcal conjugate vaccine (Fig. 1B,C), based on hospital discharge records from community hospitals. Dataset 2 dated back to 1920–1923 and included incidence records for influenza and pneumonia from New York City and 4 other metropolitan centers (Fig. 1A), based on provisional weekly nationally notifiable disease surveillance reports. Finally, dataset 3 is comprised of records from 34 US Army camps during 1918 influenza pandemic (Fig. 2), taken from annual reports of the Surgeon General of the US Army. Thus, our data spanned different spatial scales (from small scale army camps, to cities and states), different time periods (1910s–1920s, 1990s–2000s), and comprised both seasonal and pandemic influenza viruses.

Our approach consisted of two separate stages: statistical inference, followed by model validation (see Methods). *Statistical Inference:* The inferential framework was based on our previously-developed approach^38^. Specifically, we formulated a transmission model of bacterial pneumonia and aimed to establish the impact of prior influenza infection. We tested three distinct hypotheses. The first, (Hypothesis 1: transmission impact), proposes that individuals co-infected with influenza and bacterial pneumonia are more infectious compared to those with no history of recent influenza infection. The second, (Hypothesis 2: susceptibility impact), proposes that infection with influenza enhances risk of developing bacterial pneumonia. The third, (Hypothesis 3: pathogenesis impact), proposes that influenza infection only affects the severity of subsequent bacterial pneumonia, without concomitant transmission consequences. We emphasize that while the hypothesized mechanisms are distinct, they are not mutually exclusive. *Model Validation:* We carried out model validation by assessing out-of-fit predictions. Specifically, we challenged the model fitted to data from New York City from 1920–1923 (dataset 2), to predict pneumonia incidence in Chicago, Philadelphia, Los Angeles and Baltimore over the same time period. Finally, we used this fitted model to predict dataset 3, from the 1918 Spanish ‘flu pandemic.

## Results

### The nature of the association

We found no evidence that influenza infection affects either the transmission (hypothesis 1) or the pathogenesis (hypothesis 3) of bacterial pneumonia in Datasets 1 (A & B), and 2 (New York City). As shown in Fig. 3A,D,G, the 95% confidence intervals for the relative transmissibility of coinfected individuals, *θ*, included the null expectation of 1 in all three time series (dataset 2: 0–5; dataset 1A: 0.38–9.5; and dataset 1B: 0–1). Similarly, Fig. 3C,F,I present the 95% confidence intervals for the relative disease severity of coinfected cases, *ξ*, which also included the null expectation of 1 (dataset 2: 0.34–1.4; dataset 1A: 0.69–19; and dataset 1B: 0.84–2.6) in all three time series. In contrast, in two of the three time series, we found evidence that influenza infection increases susceptibility (hypothesis 2) to bacterial pneumonia. In these two datasets, we estimated the relative susceptibility of coinfected individuals, *ϕ*, to be considerably larger than one—CIs for *ϕ* were (29–1100), and (59–310) for datasets 2 and 1B, respectively. In dataset 1A, however, while the maximum likelihood estimate for *ϕ* exceeded 100, the 95% confidence interval (0.3–450) included the null expectation of 1. While the impact of increased susceptibility in these data is not statistically significant, the broad confidence interval is suggestive of an effect.

**Figure 1:**
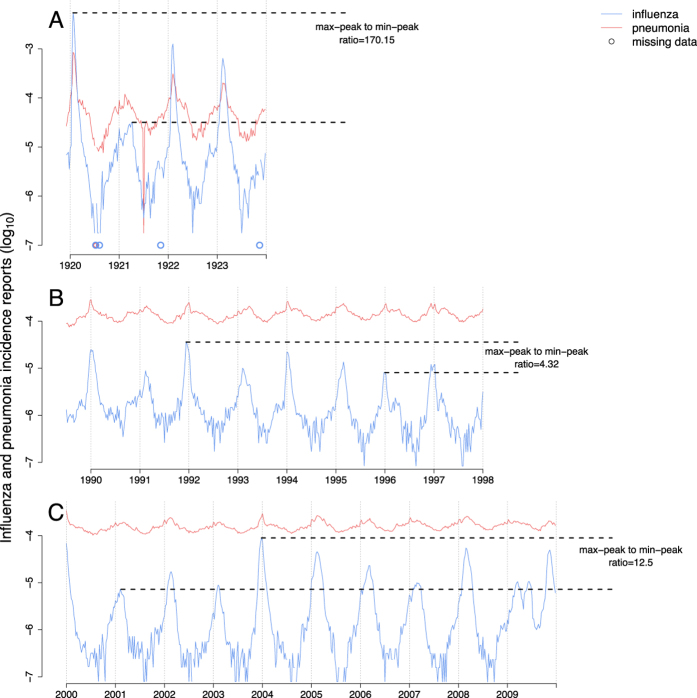
Datasets 1, and 2. (**A**) Weekly incidences of influenza and pneumonia in New York city (Dataset 2). (**B**,**C**) Weekly incidences of influenza and pneumonia in Illinois, before (dataset 1A) and after (dataset 1B) the introduction of pneumococcal conjugate vaccine (PCV), respectively. The variability in influenza in each of the datasets are presented as the ratios of largest to smallest peaks.

In Shrestha *et al.*^38^, we demonstrated, via a simulation study, that our ability to make a correct inference regarding enhanced susceptibility requires variability in seasonal influenza peaks. When influenza outbreaks from year to year are of a consistent size, the signal of any interaction is too weak to be detectable. To explore whether this accounts for the differing conclusions drawn from datasets 1A and 1B, we compared the difference in log-likelihood units between MLEs and the null expectation of *ϕ* = 1. This quantity was correlated with the magnitude of variability in influenza epidemics in the respective datasets. The variability in influenza incidence was largest in dataset 2 (Fig. 1A) and these data contained the strongest signal: the log-likelihood difference between the MLE and the null model was 25.83 log-likelihood units. The variation in influenza epidemics was the smallest in dataset 1A and we found only a 3.64 log-likelihood difference between the MLE and the null model.

A notable feature of the likelihood profiles presented in Fig. 3 is the broad confidence bounds in the susceptibility parameter, *ϕ*. To better understand this, we constructed two-dimensional likelihood profiles. The large confidence intervals in *ϕ* could be partially attributed to (i) a trade-off between the three different hypotheses (as visible in the likelihood surfaces in Fig. S-6, Supplementary Materials), and (ii) the uncertainty resulting from the need to simultaneously estimate the reporting ratio for influenza, suggested by the trade-off between *ϕ* and 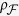 in the likelihood surface (Fig. S-9, Supplementary Materials).

We also estimated the time scale of interaction by examining susceptibility impact among those infected with influenza more than one (*ϕ*_1_) or two (*ϕ*_2_) weeks prior. As we show in Figure S-5 of the supplementary materials, we cannot reject the null hypothesis that *ϕ*_1_ = *ϕ*_2_ = 1, indicating that enhanced susceptibility to pneumonia following influenza infection operates over a short time period. These findings are consistent with results of challenge experiments in animal models, which have concluded that the influenza-pneumococcal interaction operates over a short window—only instances where bacterial infection lags viral infection by 5–7 days lead to increased susceptibility^10^. Immuno-kinetic models of this viral-bacterial interaction also predict a similarly short time window^11^,^39^.

Because of the consistency of these findings with those of Shrestha *et al.*^38^, we assessed whether the impact of influenza we detected here is limited to pneumococcal pneumonia. To do so, we first generated predicted pneumonia incidence excluding pneumococcal pneumonia by subtracting the predicted pneumococcal pneumonia incidence (using our previous model^38^), from predicted pneumonia incidence. These model predictions were then compared against the Illinois data (dataset 1). We found that predictions of the MLE models, which included susceptibility impact of influenza on bacterial pneumonia, were superior to the null models: R^2^ goodness of fit for the MLE model were 0.532 and 0.745 for pre- and post-vaccine Illinois data, compared to 0.471 and 0.697 for the null model. (See Figures S-14 and S-15 in the Supplementary Materials for comparisons of pre- and post-PCV Illinois data.) This suggests that the interaction between influenza and bacterial pneumonia we have detected cannot be entirely attributed to the interaction between influenza and pneumococcal pneumonia.

### The intensity of the susceptibility impact

Without evidence to support hypotheses 1 and 3 in all three time series, we re-estimated the susceptibility impact of influenza on pneumonia assuming *θ* = 1 and *ξ* = 1. The susceptibility impact, *ϕ*, was estimated to be 80 (CI: 25–150) in dataset 2 (Fig. 3B: inset) and 110 (CI: 63–240) in dataset 1B (Fig. 3H: inset). In dataset 1A (Fig. 3E: inset), the susceptibility hypothesis was marginally significant with the 95% confidence interval for *ϕ* extended from 1.2 to 410. Given that this cut-off is within the range of variability introduced in fitting a profile for 95% confidence interval, we interpret this to be a weak signal for presence of an interaction.

To check the consistency of these findings, we independently estimated the susceptibility impact, *ϕ*, in 4 other cities, namely Chicago, Baltimore, Philadelphia and Los Angeles, from 1920 to 1923. The respective city-specific likelihood profiles of *ϕ* [Fig. 4B] arrive at MLEs that are surprisingly consistent, ranging from 75 to 135. These results suggest that recent influenza infection enhances the susceptibility to pneumonia by a factor of ~100.

### Model validation during non-pandemic periods

To assess the predictive ability of our model, we challenged the model fitted to New York City data (dataset 1) to predict pneumonia incidence in Chicago, Philadelphia, Baltimore and Los Angeles, using the city-specific population size and influenza incidence as covariates. These predictions, henceforth referred to as out-of-fit predictions, were based on the MLE pneumonia parameters estimated from New York City data, and importantly not fit to the city-specific pneumonia incidence. We compared the *R*^2^ goodness of fit of the predictions made by the fitted model, against the best-fit null model (*ϕ* = 1). As shown in Fig. 4A, the MLE-model generated quantitatively good out-of-fit predictions with *R*^2^ values of 0.85, 0.48 and 0.8 for three out of four cities, which were superior to those generated by the null-model. The MLE-based prediction in Los Angeles was inferior to the null-model prediction (the negative value indicates the prediction was also inferior to predictions based on the mean alone). Comparisons of out-of-fit predictions from both models and the data (Fig. S-8 in the Supplementary Materials), show that MLE-based prediction overestimated the winter peak in 1920 for Los Angeles, which had an unusually low peak compared to the other cities that winter.

### Model validation during the 1918 pandemic

**Figure 2:**
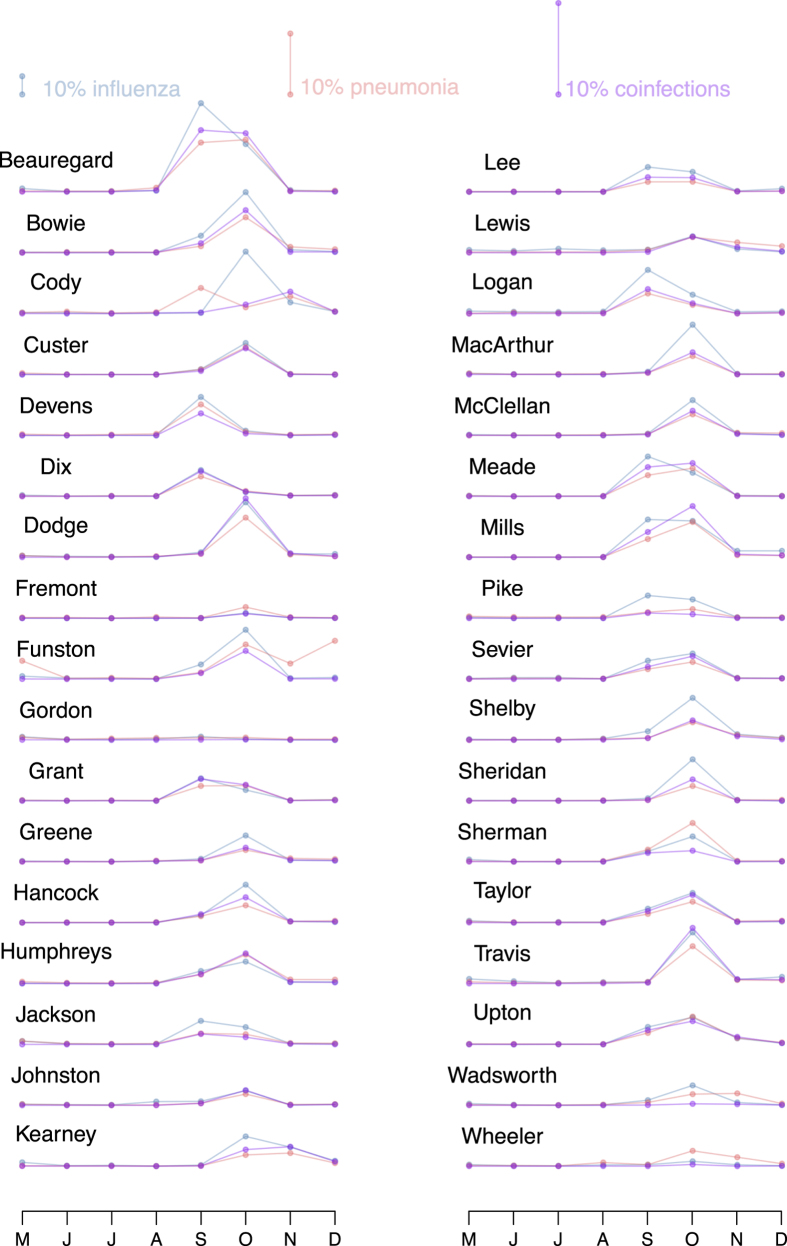
Dataset 3. The figure shown monthly incidences of influenza (blue), pneumonia (red), and coinfections (purple) across 34 US Army camps spanning 8 months, from May of 2018 to Dec 1918. This covers the fall wave in 1918. The vertical colored lines indicate the scale of the graph where the length indicates incidence of 10%.

**Figure 3:**
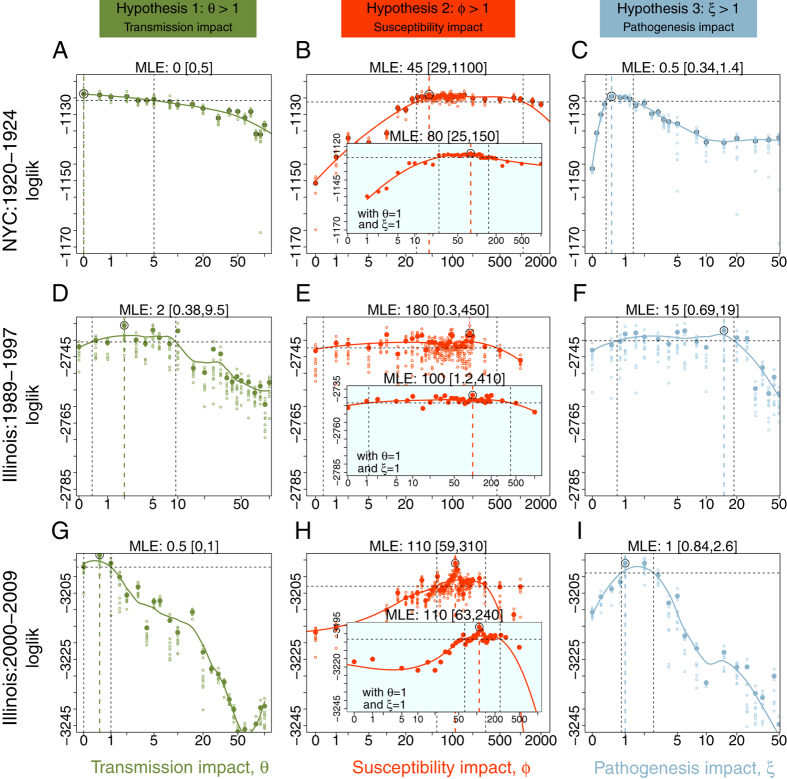
The nature and intensity of interactions between influenza and pneumonia. The nature and intensity of interactions between influenza and pneumonia, inferred in (**A**–**C**) New York City from 1920 to 1924 (dataset 2); and in the state of Illinois before the introduction of PCV from (**D**–**F**) 1990 to 1997 (dataset 1A); and after the introduction of PCV from (**G**–**I**) 2000 to 2009 (dataset 1B). Arranged column-wise are the tests for the three hypotheses, hypothesis 1 (transmission impact), hypothesis 2 (susceptibility impact), and hypothesis 3 (pathogenesis impact). Plotted in each graph are likelihood profiles for the respective parameters—the profiles are created by fitting a smooth line through the log of the arithmetic mean likelihoods (shown in large dots) in 10 repeated likelihood estimates (shown in small dots). The values within the two dashed black lines are within the estimated 95% confidence interval, and the value marked with dashed colored line represents the maximum likelihood estimate (MLE). For each of the three parameters, value of 1 represents the null hypothesis. For hypothesis 2, we show the profiles with *θ* = 1, and *ξ* = 1 (i.e. after rejecting hypotheses 1 and 3) in the inset graphs.

**Figure 4:**
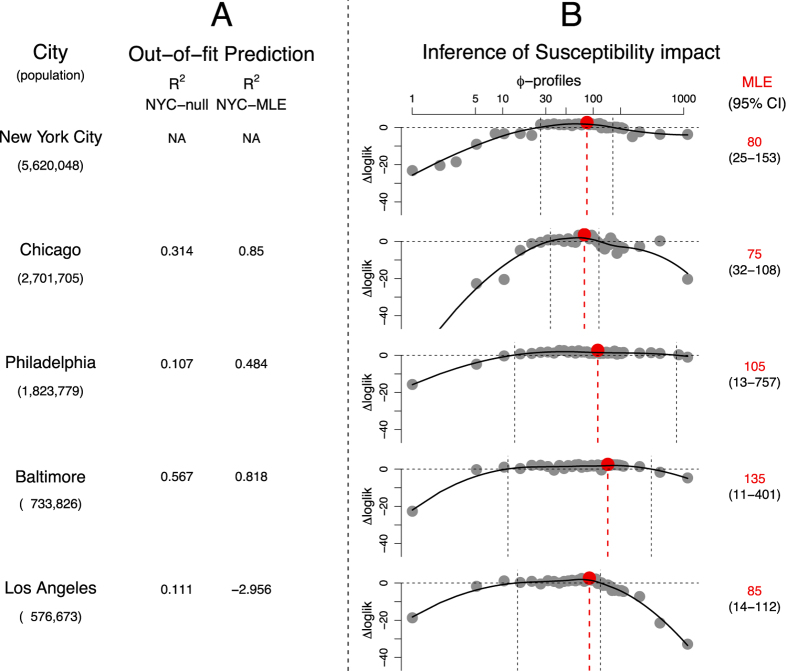
Susceptibility impact of influenza on pneumonia in four cities between 1920–1923. We examine the susceptibility impact in four cities, Chicago, Philadelphia, Baltimore and Los Angeles in two different ways. (**A**) First, we analyze out-of-fit predictions in the four cities, using the null (*ϕ* = 1) and the MLE (*ϕ* = 80) model arising from the New York City data. Presented are *R*^2^ goodness of fits for both models for each of the cities. We do not present *R*^2^ goodness of fits for New York because New York data were used in constructing the MLE model. [See Fig. S-7 in SOM for comparisons of the data and the predictions.] (**B**) Second, we independently estimate the susceptibility impact, *ϕ*, from each of the 4 datasets, following the same procedure used for New York City data. Presented are the likelihood profiles, and the 95% confidence intervals.

**Figure 5:**
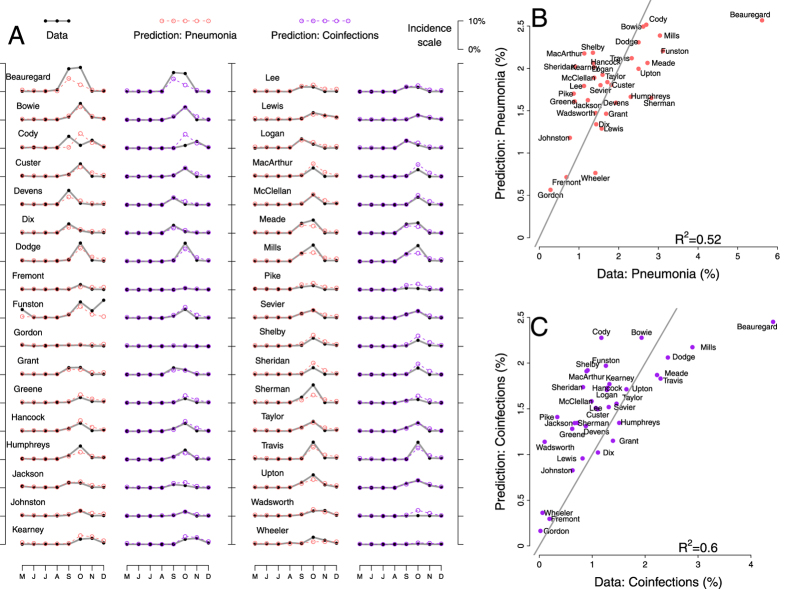
Prediction of pneumonia and coinfections across 34 US Army Camps during the Fall wave of 1918 influenza pandemic. We predict monthly pneumonia incidence (fraction of the camp population reported with pneumonia, shown in red) and monthly coinfection incidence (fraction of the camp population reported with both influenza and pneumonia, shown in purple) over a period of 8 months covering the fall wave of 1918 pandemic, across 34 camps. (**A**) Shown are comparisons of model predictions (which are forward simulations of the MLE^+^ model, averaged over 1000 simulations per camp) and the data for each of the 34 camps. Comparisons of the predictions of pneumonia (**B**) and coinfections (**C**) during the 1918 fall pandemic over 34 US Army camps, with the data during the data period. Presented are average monthly incidences (%) during the 3 months (S,O,N) spanning the fall pandemic. The *R*^2^ goodness of fit between the data and the prediction were 0.52 and 0.6 for pneumonia predictions and coinfection predictions, respectively.

For each of the 34 army camps, we predicted monthly cases of pneumonia and coinfections for 8 months spanning the fall wave of the 1918 influenza pandemic. The predictions were based on MLE^+^ model (see Methods for details on MLE^+^ model), and utilized the monthly influenza case reports in each of the camps (Fig. 2). The predictions captured substantial amounts of variability in both the incidence of pneumonia (Fig. 5A, in red) and the frequency of coinfections (Fig. 5A, in purple), as well as the timing of their peaks across camps. The *R*^2^ goodness of fit between the data and the predictions (34 camps × 8 months) were 0.61 for pneumonia predictions and 0.7 for coinfection predictions. Camp-specific predictions of the size of the pneumonia outbreaks and coinfections during the 3 months spanning the fall pandemic (September, October, and November of 1918) were also in fair agreement with the data. The *R*^2^ goodness of fit were 0.52 and 0.6 for pneumonia predictions and coninfection predictions, respectively (See Fig. 5B,C). The predictions were fairly robust to variations in turnover rate and reporting ratios (See Fig. S-12 in the supplementary materials).

## Discussion

By examining hospitalization reports of influenza and pneumococcal pneumonia in Illinois over the past two decades, we had previously reported that influenza infection enhanced susceptibility to pneumococcal pneumonia by ~100 fold up to a week^38^. We had also found that the interaction was limited to susceptibility enhancement, with no evidence for either a transmission or pathogenesis impact. Here, we turned our attention to the consequences of influenza infection on bacterial pneumonia, more broadly. Additionally, the data we analyzed here address the consequences of both seasonal and pandemic influenza. These data differed in their spatial resolution (ranging from army camps to cities to state level), and spanned different time periods with very different prevention and treatment practices, including the use of influenza and pneumococcal vaccines, as well as access to antibiotics. Our findings here are very consistent with those of Shrestha *et al.*^38^. Again, we are led to conclude that the data support increased susceptibility by a factor of ~100 and were able rule out hypothesized transmission and pathogenesis impacts in all time series we examined. Furthermore, our results suggest that interaction between influenza and bacterial pneumonia is not limited to interaction with pneumococcal pneumonia alone.

While the interaction strength is considerable at the level of the individual, its numerical effect at the population level is rather modest, accounting for 2–7% of pneumonia cases in New York City between 1920 and 1924, and less that 2% of pneumonia cases in Illinois between 1990–1998 and 2000–2010. [Please refer to Fig. S-9 in the supplementary materials for influenza attributable etiological fraction of pneumonia.] The ability to infer the mechanism and magnitude of this process reflects the potential usefulness of combining mechanistic models with likelihood-based inference methods. The key to the detectability of the signal—i.e., the source of the information harvested—is the interannual variability in influenza peaks^38^. Consequently, we were not able to detect an interaction in the dataset with substantially smaller variability in influenza incidence. While the strength of the signal of the interaction may have varied across datasets, the estimated intensity of the susceptibility impact was remarkably consistent.

In our model validation analyses, we aimed to assess how much of the variability in the pneumonia incidence data could be explained by the hypothesized mechanism of interaction. This was accomplished by analyzing out-of-fit predictions made in two different time periods, during the 1918 pandemic across 34 army camps and after the pandemic in 4 different cities. The scale of influenza epidemics varied drastically between the two datasets, yet the predictions based on the same hypothesis were able to explain a considerable amount of variability in the data. Previous work, most of which has focused on individual autopsy reports, has detailed the extent of the interaction between influenza and bacterial pneumonia during the 1918 pandemic^26^,^27^,^40^,^41^,^42^. Our results gleaned from epidemiological reports are consistent with these findings and are furthermore very similar to coinfection dynamics during non-pandemic periods.

It is important to point out that our work does not necessarily rule out potential variability in the the intensity of interaction between influenza and bacterial pneumonia at different times and places. Different influenza subtypes may elicit varying degrees of immune response, which may lead to variability in the interaction with bacterial pneumonia^36^. Variability in the interaction can also be introduced by how influenza may interact with different bacterial agents that contribute to bacterial pneumonia. Risk of invasive pneumonia has been shown to be serotype-dependent for pneumococcus^43^, and there is potential for similar serotype level variability in the interactions with influenza^37^. However, what our results do suggest is that the scale of the manifestation of bacterial coinfections during the 1918 pandemic, as observed in the army camp hospitalization reports, does not necessarily imply that interaction between pandemic influenza strain and bacterial pneumonia was abnormally higher. This is consistent with the finding in animal models that coinfection with H1N1 influenza strain expressing 1918 proteins are not significantly different from a strain that does not, in terms of the subsequent bacterial loads and within-host kinetics^44^. Further research is merited to quantify variability in coinfection dynamics resulting from different strain of influenza.

We have tried to be both pragmatic and parsimonious in our modeling choices, guided by the availability of data, and general understanding of transmission dynamics. We have assumed that the transmission of bacterial pneumonia is homogenous, and have not included heterogeneities arising from age-dependent contact patterns or associated risk factors. It would be particularly important to know how the interactions may differ in age groups, especially children where the burden is large. We have also assumed the bacterial carriage to be constant: we were unable to incorporate a mechanistic model in the absence of a clear understanding of causal links between carriage and transmission.

Our work is based on hospitalization reports and categorization of influenza and pneumonia based on the standard of practice at different times and settings, which may have been variable between different datasets. Of note, the influenza virus itself was only discovered in 1930s^45^, more than a decade after the 1918 pandemic. Hence the categorizations, especially for the earlier datasets, are likely to be only symptom-based and subject to variability in the diagnosis. One important known difference between dataset 1 and datasets 2 and 3 is that whereas pneumonia case reports used in dataset 1 excluded exclusively influenza viral pneumonia cases, such sub-categorizations were not available for pneumonia case reports in datasets 2 and 3. Hence, pneumonia case reports in datasets 2 and 3 may also include some non-bacterial, influenza viral pneumonia cases, which has the potential to lead to overestimation of the interaction. We note, however, that the inferences we made from these data (dataset 2) were very similar to those drawn from data that excluded influenza viral pneumonia (dataset 1). More generally, other exclusively viral etiologies of pneumonia, which are difficult to diagnose, especially with the abundance of bacterial co-infections^46^, have not been distinguished in any of the pneumonia case reports used in this study. Although they could inflate the level of bacterial pneumonia, they are unlikely to systematically influence the signal of interaction. Ideal forms of data, which would include microbiological confirmation of viral and bacterial etiologies, are generally not available at the population level. Finally, the hospitalization reports also do not distinguish the severity of influenza or pneumonia infection in reported cases, and may lack requisite details to identify the impact of influenza on the severity of pneumonia: Our finding of no severity impact in hospitalization data should be interpreted in this context.

Our work suggests that the impact of influenza on pneumonia is not limited to the clinical manifestation of the infection, i.e. increasing the severity of the infection. In particular, we find a presence of an epidemiological impact of influenza on pneumonia. This suggests that interventions targeted at reducing the risk of pneumonia, either via vaccines targeted towards bacterial sources of pneumonia^47^ or against influenza, which are particularly effective in reducing the risk of subsequent risk of pneumonia^48^,^49^, are likely to be more effective compared to post hoc treatment and case management. Furthermore, given that the interaction operates at short time scales, antiviral treatment of influenza patients may not be as efficacious in preventing secondary bacterial infections^39^. On the other hand, the efficacy and effectiveness of influenza prevention strategies should perhaps also include prevention of pneumonia, thereby enhancing the value of influenza prevention^50^.

## Materials and Methods

### Data

#### Dataset 1: Weekly hospitalizations of influenza and pneumonia in the State of Illinois, 1990–1997 (before the introduction of the pneumococcal conjugate vaccine), and 2000–2009 (after the introduction of pneumococcal conjugate vaccine)

Datasets 1 (A&B) consisted of weekly hospitalization of influenza and pneumonia in the state of Illinois, between 1990 and 1997 [Fig. 1B], and between 2000 and 2009 [Fig. 1C], respectively. These data were obtained from the State Inpatient Databases (SID) of the Healthcare Cost and Utilization Project (HCUP), maintained by the Agency for Healthcare Research and Quality (AHRQ), through an active collaboration^51^. This database contains all hospital discharge records from community hospitals in the state. Cases were identified by the presence of the relevant diagnostic codes listed anywhere in the patients’ record, including influenza (487–488), or all-cause pneumonia, excluding influenza (480–486). Weekly time series were created for each disease outcome. The data are presented as incidences, using mid-year population size estimates for the state which were obtained from the United States Census Bureau. Population estimates are shown in Fig. S-1 in the supplementary materials.

#### Dataset 2: Weekly case reports of influenza and pneumonia, NYC and 4 other population centers, 1920–1923

Dataset 2 consisted of weekly case reports of influenza and pneumonia in five large population centers in the US, namely New York City, Chicago, Philadelphia, Baltimore, and Los Angeles, from 1920 to 1923. The choice of the cities was primarily made on the basis of data availability. These data were obtained from the Project Tycho database at the University of Pittsburgh (www.tycho.pitt.edu)^52^. The Project Tycho digitized data from provisional weekly nationally notifiable disease surveillance report from US cities and states between 1888 and 2011 that were published in various journals by the Centers for Disease Control and Prevention and its precursors. The pneumonia and influenza case reports used here were published in the Public Health Reports by the US Public Health Service. [See Fig. 1A for New York City data, Fig. S-1 for data on other cities, and section S-1 in supplementary online materials (SOM) for detailed information on the data.] The population sizes of each of these cities are also taken from 1920 census.

#### Dataset 3: Monthly case reports of cases of influenza, pneumonia, and coinfections across 34 US Army camps, 1917–1918

Dataset 3 [Fig. 2] consisted of monthly hospitalization reports of cases of influenza, pneumonia and both influenza and pneumonia across 34 US Army camps from May 1918 to Dec 1918. The data were obtained from the annual reports of the Surgeon General of the US Army and digitized for analyses. Influenza cases consisted of individuals reported with influenza only, influenza and bronchopneumonia, influenza and lobar-pneumonia, and influenza with other complications. Pneumonia cases consisted of individuals reported with bronchopneumonia only, lobar-pneumonia only, influenza and bronchopneumonia, and influenza and lobar-pneumonia. Finally, coinfection cases consisted of individuals that were reported with influenza and bronchopneumonia, and influenza and lobar-pneumonia. The population of the Army camps were also taken from the annual reports.

### Model of pneumonia transmission and viral-bacterial interaction

We used a previously developed modeling framework^38^, to model the underlying transmission dynamics of bacterial pneumonia and interaction between influenza and pneumonia. This model encapsulates three distinct hypotheses pertaining to the nature of the interaction between influenza and bacterial pneumonia. The first hypothesis, (**H1**: transmission impact), proposes that individuals co-infected with influenza and bacterial pneumonia are more transmissive compared to individuals that are not infected with influenza. The second hypothesis, (**H2**: susceptibility impact), proposes that infection with influenza enhances risk of developing bacterial pneumonia. The third hypothesis, (**H3**: pathogenesis impact), proposes that influenza infection increases the severity of subsequent bacterial pneumonia. The three hypotheses describe three distinct mechanisms by which epidemiological data of bacterial pneumonia are potentially affected by influenza. While the hypothesized mechanisms are distinct, they are not necessarily mutually exclusive. We do not explicitly model the transmission dynamics of influenza, and influenza weekly case reports are taken as a covariate.

The underlying transmission model is an adapted version of the well-studied *SIRS* compartmental model^53^,^54^ where the host population is divided into 3 compartments according to their infection status with respect to bacterial pneumonia—(i) *S* consists of susceptible hosts; (ii) *I* consists of hosts who are currently infected; and (iii) *R* consists of hosts that have recently recovered from an infection. Susceptibles experience a *per capita* hazard of bacterial pneumonia, *λ*(*t*), that is modeled to consist of two sources of transmission: (i) individuals that are currently infectious with bacterial pneumonia, *I*(*t*), and (ii) bacterial carriage in the population that is constant and not reflected in the incidence, *ω*, yielding: 
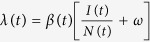
. Here, *β*(*t*) represents seasonally varying transmission rate. Individuals remain infectious for an average duration of 1/*γ*, and the protection imparted by a previous infection lasts for 1/*ε* on average.

In order to incorporate the three hypothesized mechanisms of the effect of influenza on bacterial pneumonia within this framework, compartments *S* and *I* are further divided into sub-compartments, depending on their status with respect to the influenza infection. In particular, sub-compartments 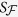 and 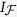 consist of susceptible and infectious hosts who were recently infected with influenza, and sub-compartments *S*_*U*_ and *I*_*U*_ of hosts who were not. We estimate the size of 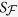, assuming that it is proportional to the current incidence of influenza corrected for underreporting. Hence, at time *t*, 
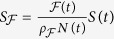
, where *N*(*t*) is the size of the population, 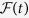 is number of influenza cases reports, and 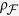 is the reporting probability.

To formulate hypothesis 1, we distinguish between the transmission contributions of those infected with bacterial pneumonia according to their status with respect to influenza. In particular, we assume that transmission potential of individuals recently infected with influenza is modulated by a factor *θ* compared to those uninfected with influenza, such that: 
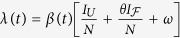
. Thus, hypothesis 1 is formulated as *θ* > 1, with the null hypothesis given by *θ* = 1. To formulate hypothesis 2, we hypothesize that susceptibles in the two sub-compartments, *S*_*S*_, and 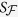 experience different hazard rates, *λ*(*t*), and 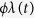, respectively. The hazard ratio *ϕ* is taken as the measure of susceptibility impact due to influenza infection. Again, hypothesis 2 is formulated as *ϕ > *1, with the null hypothesis *ϕ = *1. Finally, to formulate hypothesis 3, we hypothesize that individuals coinfected with influenza develop more severe symptoms, and are likely to be reported at a higher rate, i.e. *ξ* times more than individuals not infected with influenza. Hypothesis 3 is formulated as *ξ* > 1, with *ξ* = 1 being the null. The model and the three hypothesized mechanisms of interactions are schematically presented in Fig. S.4 in the supplementary materials.

### Hypothesis testing and inference of the intensity of the interaction

The three central hypotheses concerning the nature of the interaction are tested using a likelihood based inference approach. We utilized the framework of partially observed Markov processes^55^,^56^,^57^,^58^, that is implemented in a freely available software package pomp^59^. In this framework, the model in conjunction with a set of parameters Θ = {Θ_1_,…,Θ_*k*_} is confronted with data, *y*(*t*_*j*_), *j* = 1,…, *n* to come up with a likelihood estimate 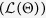. The evidence that a focal parameter Θ_*i*_ takes a value *p*, i.e. Θ_*i*_ = *p*, is reflected in the maximum of all likelihood estimates with the chosen parameter fixed at *p*, and all other parameters varied: 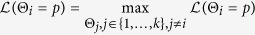. Now, by considering likelihoods across a range of value *p*_1_,…, *p*_*m*_, a likelihood profile for the focal parameter Θ_*i*_ is constructed. The profile describes the strength of evidence pertaining to the focal parameter over the profiled range. The value corresponding to the largest likelihood estimate is considered a maximum likelihood estimate (MLE), and values corresponding to the likelihood estimates 1.98 units below maximum are taken to be 95% confidence interval.

We constructed likelihood profiles for each of the three hypothesis-related parameters, *θ*, *ϕ*, and *ξ*, separately for dataset 1 (A&B), and dataset 2 (New York City). Additionally, for the remaining portion of dataset 2 (Chicago, Philadelphia, Baltimore, and Los Angeles), we constructed likelihood profile for *ϕ*. For each of the hypothesis, if the null value of 1 was contained within the 95% confidence interval, then the hypothesis was rejected and the MLE was taken to be the point estimate of the intensity of the interaction of the hypothesized nature.

### Prediction Model

To assess the predictability of the inferred model and the nature and intensity of the interaction, we developed prediction models. The aim of the prediction models was to predict the number of pneumonia cases in a specific location over a period of time, on the basis of hospitalization reports of influenza cases, and previously identified model that was most consistent with the data, i.e. the MLE model. In particular, the prediction model were not directly fit to the data on which the predictions were made.

Two prediction models were developed. The first prediction model aimed to predict pneumonia cases during non-pandemic period in dataset 2 (Chicago, Philadelphia, Baltimore, and Los Angeles). Since these predictions were at the scale of city, we simply used the MLE model inferred from dataset 2 (New York City) as the prediction model. The set of parameter values that characterize the MLE model are presented in Table S2 in the supplementary materials. The second prediction model aimed to predict pneumonia cases in dataset 3 — 34 US Army camps during the Fall wave of the 1918 influenza pandemic. We used the same MLE model but with changes to 3 parameters, (i) birthrate *μ*, (ii) reporting ratio for influenza 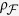, and (iii) reporting ratio for pneumonia, *ρ*_*p*_ (referred to as MLE^+^). Since birth events would not be relevant for US Army camps, *μ* was instead interpreted to represent rate of turnover in camps. This was taken to be 0.25 per year, ie an individual would reside in a camp for 4 years on average. The two reporting rates were allowed to change to account for differences in reporting practices between a city compared to an army camp. We explored a range of possible reporting ratios for pneumonia and influenza, and picked the reporting ratios that yielded the best predictions. They were 50% for pneumonia and 30% for influenza. Sensitivity of the predictions to the turnover rate and reporting ratios are shown in the supplementary materials.

## Additional Information

**How to cite this article**: Shrestha, S. *et al.* The role of influenza in the epidemiology of pneumonia. *Sci. Rep.* **5**, 15314; doi: 10.1038/srep15314 (2015).

## Supplementary Material

Supplementary materials

## References

[b1] WHO. World health organization. global health estimates: Disease burden. http://www.who.int/healthinfo/global_burden_disease/estimates_regional_2000_2011/en/index1.html (last accessed: March 20, 2015) (2013).

[b2] Lozano R. *et al.* Global and regional mortality from 235 causes of death for 20 age groups in 1990 and 2010: a systematic analysis for the global burden of disease study 2010. The Lancet 380, 2095–2128 (2013). 10.1016/S0140-6736(12)61728-0 PMC10790329 23245604

[b3] Bosch A. A. T. M., Biesbroek G., Trzcinski K., Sanders E. A. M. & Bogaert D. Viral and bacterial interactions in the upper respiratory tract. PLoS Pathog. 9, e1003057 (2013). 23326226 10.1371/journal.ppat.1003057 PMC3542149

[b4] Weinberger D. M., Klugman K. P., Steiner C. A., Simonsen L. & Viboud C. Association between Respiratory Syncytial Virus Activity and Pneumococcal Disease in Infants: A Time Series Analysis of US Hospitalization Data. PLoS Med. 12, e1001776 (2015). 25562317 10.1371/journal.pmed.1001776 PMC4285401

[b5] Rudan I., Boschi-Pinto C., Biloglav Z., Mulholland K. & Campbell H. Epidemiology and etiology of childhood pneumonia. Bulletin of the World Health Organization 86, 408–416B (2008). 18545744 10.2471/BLT.07.048769 PMC2647437

[b6] Falsey A. R. *et al.* Bacterial complications of respiratory tract viral illness: A comprehensive evaluation. Journal of Infectious Diseases 208, 432–441 (2013). arXiv: http://jid.oxfordjournals.org/content/208/3/432.full.pdf+html. 23661797 10.1093/infdis/jit190 PMC3699009

[b7] McCullers J. A. Do specific virus–bacteria pairings drive clinical outcomes of pneumonia? Clinical Microbiology and Infection 19, 113–118 (2013). 23231363 10.1111/1469-0691.12093

[b8] McCullers J. A. Insights into the interaction between influenza virus and pneumococcus. Clin. Microbiol. Rev. 19, 571–582 (2006). 16847087 10.1128/CMR.00058-05 PMC1539103

[b9] McCullers J. A. The co-pathogenesis of influenza viruses with bacteria in the lung. Nat Rev Micro 12, 252–262 (2014). 10.1038/nrmicro3231 24590244

[b10] McCullers J. A. & Rehg J. E. Lethal synergism between influenza virus and *Streptococcus pneumoniae*: Characterization of a mouse model and the role of platelet-activating factor receptor. J. Infect. Dis. 186, 341–350 (2002). 12134230 10.1086/341462

[b11] Sun K. & Metzger D. M. Inhibition of pulmonary antibacterial defense by interferon-*γ* during recovery from influenza infection. Nat. Med. 14, 558–564 (2008). 18438414 10.1038/nm1765

[b12] Kukavica-Ibrulj I. *et al.* Infection with human metapneumovirus predisposes mice to severe pneumococcal pneumonia. J. Virol. 83, 1341–1349 (2009). 19019962 10.1128/JVI.01123-08 PMC2620891

[b13] Shahangian A. *et al.* Type I IFNs mediate development of postinfluenza bacterial pneumonia in mice. J. Clin. Invest. 119, 1910–1920 (2009). 19487810 10.1172/JCI35412 PMC2701856

[b14] van der Sluijs K. *et al.* IL-10 is an important mediator of the enhanced susceptibility to pneumococcal pneumonia after influenza infection. Journal of Immunology 172, 7603–7609 (2004). 10.4049/jimmunol.172.12.7603 15187140

[b15] Peltola V. & McCullers J. Respiratory viruses predisposing to bacterial infections: role of neuraminidase. The Pediatric infectious disease journal 23, S87 (2004). 14730275 10.1097/01.inf.0000108197.81270.35

[b16] Avadhanula V. *et al.* Respiratory viruses augment the adhesion of bacterial pathogens to respiratory epithelium in a viral species- and cell type-dependent manner. Journal of Virology 80, 1629–1636 (2006). arXiv: http://jvi.asm.org/content/80/4/1629.full.pdf+html. 16439519 10.1128/JVI.80.4.1629-1636.2006 PMC1367158

[b17] Lee L. N. *et al.* A mouse model of lethal synergism between influenza virus and *Haemophilus influenzae*. The American Journal of Pathology 176, 800–811 (2010). 20042666 10.2353/ajpath.2010.090596 PMC2808086

[b18] Abramson J. *et al.* Inhibition of neutrophil lysosome-phagosome fusion associated with influenza virus infection *in vitro*. role in depressed bactericidal activity. Journal of Clinical Investigation 69, 1393–1397 (1982). 7085879 10.1172/JCI110580 PMC370213

[b19] Lee M. *et al.* A postinfluenza model of *Staphylococcus aureus* pneumonia. *The* Journal of Infectious Diseases 201, 508–515 (2010). 20078212 10.1086/650204 PMC3664424

[b20] Small C. *et al.* Influenza infection leads to increased susceptibility to subsequent bacterial superinfection by impairing NK cell responses in the lung. The Journal of Immunology 184, 2048–2056 (2010). 20083661 10.4049/jimmunol.0902772

[b21] Iverson A. *et al.* Influenza virus primes mice for pneumonia from staphylococcus aureus. The Journal of Infectious Diseases 203, 880–888 (2011). 21278211 10.1093/infdis/jiq113 PMC3071123

[b22] Broug-Holub E. *et al.* Alveolar macrophages are required for protective pulmonary defenses in murine klebsiella pneumonia: elimination of alveolar macrophages increases neutrophil recruitment but decreases bacterial clearance and survival. Infection and Immunity 65, 1139–1146 (1997). 9119443 10.1128/iai.65.4.1139-1146.1997 PMC175109

[b23] Rehm S., Gross G. & Pierce A. Early bacterial clearance from murine lungs. Species-dependent phagocyte response. Journal of Clinical Investigation 66, 194–199 (1980). 6995480 10.1172/JCI109844 PMC371698

[b24] van der Sluijs K., van der Poll T., Lutter R., Juffermans N. & Schultz M. Bench-to-bedside review: Bacterial pneumonia with influenza-pathogenesis and clinical implications. Critical Care 14, 219 (2010). 20459593 10.1186/cc8893 PMC2887122

[b25] Mina M. J. & Klugman K. P. The role of influenza in the severity and transmission of respiratory bacterial disease. The Lancet Respiratory Medicine (2014). 10.1016/S2213-2600(14)70131-6 PMC4823014 25131494

[b26] Morens D., Taubenberger J. & Fauci A. Predominant role of bacterial pneumonia as a cause of death in pandemic influenza: Implications for pandemic influenza preparedness. J. Infect. Dis. 198, 962–970 (2008). 18710327 10.1086/591708 PMC2599911

[b27] Brundage J. Interactions between influenza and bacterial respiratory pathogens: implications for pandemic preparedness. Lancet Infect. Dis. 6, 303–312 (2006). 16631551 10.1016/S1473-3099(06)70466-2 PMC7106411

[b28] Louria D., Blumenfeld H., Ellis J., Kilbourne E. & Rogers D. Studies on influenza in the pandemic of 1957-1958. II. pulmonary complications of influenza. J. Clin. Invest. 38, 213–265 (1959). 13620784 10.1172/JCI103791 PMC444127

[b29] Robertson L., Caley J. & Moore J. Importance of *Staphylococcus aureus* in pneumonia in the 1957 epidemic of influenza A. The Lancet 272, 233–236 (1958). 10.1016/s0140-6736(58)90060-6 13564806

[b30] Lindsay M. Jr, Herrmann E. Jr, Morrow G. Jr & Brown A. Jr Hong Kong influenza. Clinical, microbiologic, and pathologic features in 127 cases. JAMA 214, 1825–32 (1970). 5537337 10.1001/jama.214.10.1825

[b31] Schwarzmann S., Adler J., Sullivan R. Jr & Marine W. Bacterial pneumonia during the Hong Kong influenza epidemic of 1968-1969. Arch. Intern. Med. 127, 1037–1041 (1971). 5578560

[b32] Weinberger D. *et al.* Impact of the 2009 influenza pandemic on pneumococcal pneumonia hospitalizations in the United States. J. Infect. Dis. 205, 458–465 (2012). 22158564 10.1093/infdis/jir749 PMC3276240

[b33] Walter N. *et al.* Influenza circulation and the burden of invasive pneumococcal pneumonia during a non-pandemic period in the United States. Clin. Infect. Dis. 50, 175–183 (2010). 20014948 10.1086/649208

[b34] Kuster S. P. *et al.* Evaluation of coseasonality of influenza and invasive pneumococcal disease: Results from prospective surveillance. PLoS Med. 8, e1001042 (2011). 21687693 10.1371/journal.pmed.1001042 PMC3110256

[b35] Toschke A. *et al.* No temporal association between influenza outbreaks and invasive pneumococcal infections. Arch. Dis. Child. 93, 218–220 (2008). 17405858 10.1136/adc.2006.098996

[b36] Smith A. M. *et al.* Effect of 1918 PB1-F2 expression on influenza A virus infection kinetics. PLoS Computational Biology 7, e1001081 (2011). 21379324 10.1371/journal.pcbi.1001081 PMC3040654

[b37] McCullers J. A. *et al.* Influenza enhances susceptibility to natural acquisition of and disease due to *Streptococcus pneumoniae* in ferrets. J. Infect. Dis. 202, 1287–1295 (2010). 20822454 10.1086/656333 PMC3249639

[b38] Shrestha S. *et al.* Identifying the interaction between influenza and pneumococcal pneumonia using incidence data. Science Translational Medicine 5, 191ra84 (2013). arXiv: http://stm.sciencemag.org/content/5/191/191ra84.full.pdf. 10.1126/scitranslmed.3005982 PMC4178309 23803706

[b39] Shrestha S. *et al.* Time and dose-dependent risk of pneumococcal pneumonia following influenza: a model for within-host interaction between influenza and *Streptococcus pneumoniae*. Journal of The Royal Society Interface 10, 20130233 (2013). arXiv: http://rsif.royalsocietypublishing.org/content/10/86/20130233.full.pdf+html. 10.1098/rsif.2013.0233 PMC3730679 23825111

[b40] Soper G. The pandemic in the army camps. JAMA 71, 1899 (1918). 10.1126/science.48.1245.451 17755433

[b41] Chien Y.-W., Klugman K. P. & Morens D. M. Bacterial pathogens and death during the 1918 influenza pandemic. New England Journal of Medicine 361, 2582–2583 (2009). PMID: 20032332, arXiv: http://www.nejm.org/doi/pdf/10.1056/NEJMc0908216. 20032332 10.1056/NEJMc0908216

[b42] Klugman K. P., Chien Y. & Madhi S. A. Pneumococcal pneumonia and influenza: A deadly combination. Vaccine 27S, C9–C14 (2009). 19683658 10.1016/j.vaccine.2009.06.007

[b43] Weinberger D. M. *et al.* Association of serotype with risk of death due to pneumococcal pneumonia: A meta-analysis. Clin. Infect. Dis. 51, 692–699 (2010). 20715907 10.1086/655828 PMC2927802

[b44] Smith A. M. *et al.* Kinetics of coinfection with influenza a virus and *Streptococcus pneumoniae*. PLoS Pathog 9, e1003238 (2013). 23555251 10.1371/journal.ppat.1003238 PMC3605146

[b45] Francis T. Transmission of influenza by a filterable virus. Science 80, pp. 457–459 (1934). 17795179 10.1126/science.80.2081.457-a

[b46] Ruuskanen O., Lahti E., Jennings L. C. & Murdoch D. R. Viral pneumonia. The Lancet 377, 1264–1275 (2011). 10.1016/S0140-6736(10)61459-6 PMC7138033 21435708

[b47] Madhi S., Klugman K. *et al.* A role for *Streptococcus pneumoniae* in virus-associated pneumonia. Nature medicine 10, 811–813 (2004). 10.1038/nm1077 PMC7095883 15247911

[b48] Mina M. J., Klugman K. P. & McCullers J. A. Live attenuated influenza vaccine, but not pneumococcal conjugate vaccine, protects against increased density and duration of pneumococcal carriage after influenza infection in pneumococcal colonized mice. Journal of Infectious Diseases 208, 1281–1285 (2013). arXiv: http://jid.oxfordjournals.org/content/208/8/1281.full.pdf+html. 23852122 10.1093/infdis/jit317 PMC6281400

[b49] Katsura H. *et al.* A bivalent vaccine based on a replication-incompetent influenza virus protects against streptococcus pneumoniae and influenza virus infection. Journal of Virology (2014). arXiv: http://jvi.asm.org/content/early/2014/09/04/JVI.01205-14.full.pdf+html. 10.1128/JVI.01205-14 PMC4249093 25210171

[b50] Yamin D., Balicer R. D. & Galvani A. P. Cost-effectiveness of influenza vaccination in prior pneumonia patients in israel. Vaccine 32, 4198–4205 (2014). 24930716 10.1016/j.vaccine.2014.05.015 PMC4077912

[b51] HCUP SID Database Documentation. Healthcare Cost and Utilization Project (HCUP), 2015. Agency for Healthcare Research and Quality, Rockville, MD. http://www.hcup-us.ahrq.gov/db/state/siddbdocumentation.jsp

[b52] Van Panhuis W. G. *et al.* Contagious diseases in the united states from 1888 to the present. New England Journal of Medicine 369, 2152–2158 (2013). PMID: 24283231, arXiv: http://www.nejm.org/doi/pdf/10.1056/NEJMms1215400. 24283231 10.1056/NEJMms1215400 PMC4175560

[b53] Anderson R. M. & May R. M. Infectious Diseases of Humans; Dynamics and Control (Oxford University Press, Oxford, 1991).

[b54] Keeling M. & Rohani P. Modelling Infectious Diseases (Princeton University Press, Princeton, 2008).

[b55] Ionides E. L., Bretó C. & King A. A. Inference for nonlinear dynamical systems. Proc. Natl. Acad. Sci. USA. 103, 18438–18443 (2006). 17121996 10.1073/pnas.0603181103 PMC3020138

[b56] Ionides E. L., Breto C. & King A. A. Modeling Disease Dynamics: Cholera as a Case Study, chap. 8, 13–140 (Wiley, Hoboken NJ, 2007).

[b57] King A. A., Ionides E. L., Pascual M. & Bouma M. J. Inapparent infections and cholera dynamics. Nature 454, 877–881 (2008). 18704085 10.1038/nature07084

[b58] Shrestha S., King A. A. & Rohani P. Statistical inference for multi-pathogen systems. PLoS Comput. Biol. 7, e1002135 (2011). 21876665 10.1371/journal.pcbi.1002135 PMC3158042

[b59] King A. A. *et al.* pomp: Statistical inference for partially observed markov processes (R package). http://pomp.r.forge.r-project.org (2010).

