# Supplementary material for: The role of influenza in the epidemiology of pneumonia
Source: Sci Rep. 2015 Oct 21;5:15314. doi: 10.1038/srep15314 (PMC4614252; doi:10.1038/srep15314)
Supplement: Supplementary materials [file srep15314-s1.pdf]

Supplementary Materials for "The role of influenza on epidemiology of pneumonia"

Sourya Shrestha,[1,2,3*] Betsy Foxman,[4] Joshua Berus,[5]
Willem G. van Panhuis,[6] Claudia Steiner,[7]
Cécile Viboud,[8] Pejman Rohani[9,10,11]

[1]Department of Ecology & Evolutionary Biology, University of Michigan, Ann Arbor, MI 48109, USA
[2]Center for the Study of Complex Systems, University of Michigan, Ann Arbor, MI 48109, USA
[3]Department of Epidemiology, Johns Hopkins School of Public Health, Baltimore, MD 21205, USA
[4]Department of Epidemiology, University of Michigan, Ann Arbor, MI 48109, USA
[5]Undergraduate Research Opportunity Program, University of Michigan, Ann Arbor, MI 48109, USA
[6]Department of Epidemiology, University of Pittsburgh Graduate School of Public Health, Pittsburgh PA 15261, USA
[7]Healthcare Cost and Utilization Project, Center for Delivery, Organization and Markets,
Agency for Healthcare Research and Quality, U.S. Department of Health and Human Services, Rockville, MD 20850, USA.
[8]Division of International Epidemiology and Population Studies, National Institutes of Health, Bethesda, MD 20892, USA
[9]Odum School of Ecology, University of Georgia, Athens, GA 30602, USA
[10]Department of Infectious Diseases, School of Veterinary Medicine, University of Georgia, Athens, GA 30602, USA
[11]Fogarty International Center, National Institutes of Health, Bethesda, MD 20892, USA

*To whom correspondence should be addressed; E-mail: sourya@jhu.edu.

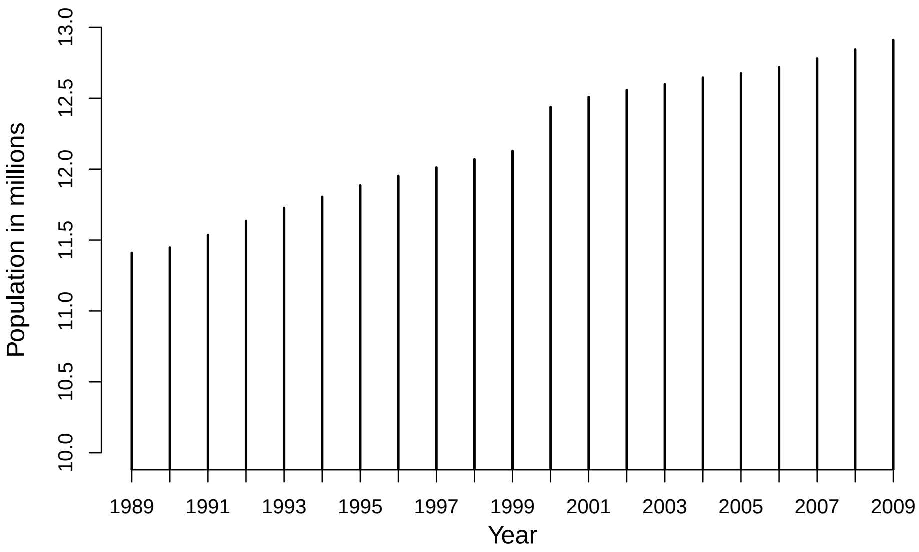

Figure S-1: Population of Illinois.

S-1 Additional Data Details

S-1.1 Dataset 1

The mid year population size estimates for the state of Illinois were obtained from the United States Census Bureau and presented Fig. S-1.

S-1.2 Dataset 2

The New York city portion of dataset 2 consists of pneumonia and influenza cases in New York city reported weekly. The dataset spans a period of 212 weeks from 1920 to the end of 1923. The time series of pneumonia case reports contained one missing observation and one outlier, and the time series of influenza case reports consisted of 4 missing observations. These missing data observations and the outlier were filled in by values obtained by linear interpolation, before the analyses were carried out. The population sizes of each of these cities are also taken from 1920 census. The population of New York city is taken from the 1920 census to be 5,620,048.

In addition, the supplementary portion of dataset 2 consist of pneumonia and influenza weekly case reports, for the same time period in 4 other cities; (i) Chicago, (ii) Philadelphia, (iii) Baltimore, and (iv) Los Angeles. The missing values in these dataset were likewise filled in using linear interpolation, before the analyses were carried out. These data are shown in Fig. S-2. The population sizes of each of these cities are also taken from 1920 census, and are shown in Fig. 3A in the main text.

S-2 Models

We adapt the $SIRS$ model[3, 4], to serve as a basis of the underlying epidemiology of bacterial pneumonia. In this framework, $S(t)$, $I(t)$, and $R(t)$ represent the numbers of susceptible, infected and recovered individuals, respectively. We further subdivide the susceptible and infected compartments into two sub-compartments

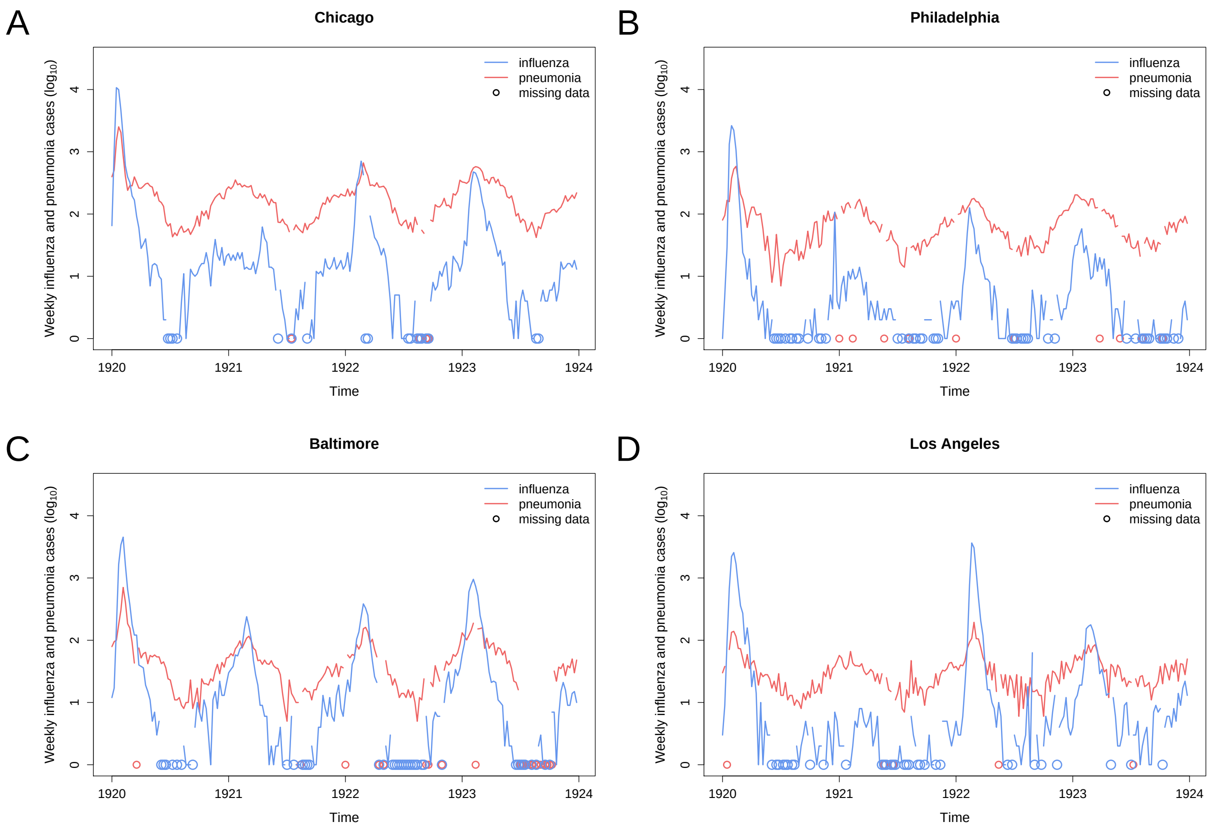

Figure S-2: Dataset 2 (Chicago, Philadelphia, Baltimore and Los Angeles.) Influenza (blue) and pneumonia cases (red), reported weekly in 4 other major US cities: (A) Chicago, (B) Philadelphia, (C) Baltimore, and (D) Los Angeles, from 1920 to 1923. The blue and red circles represent missing data for influenza and pneumonia, respectively.

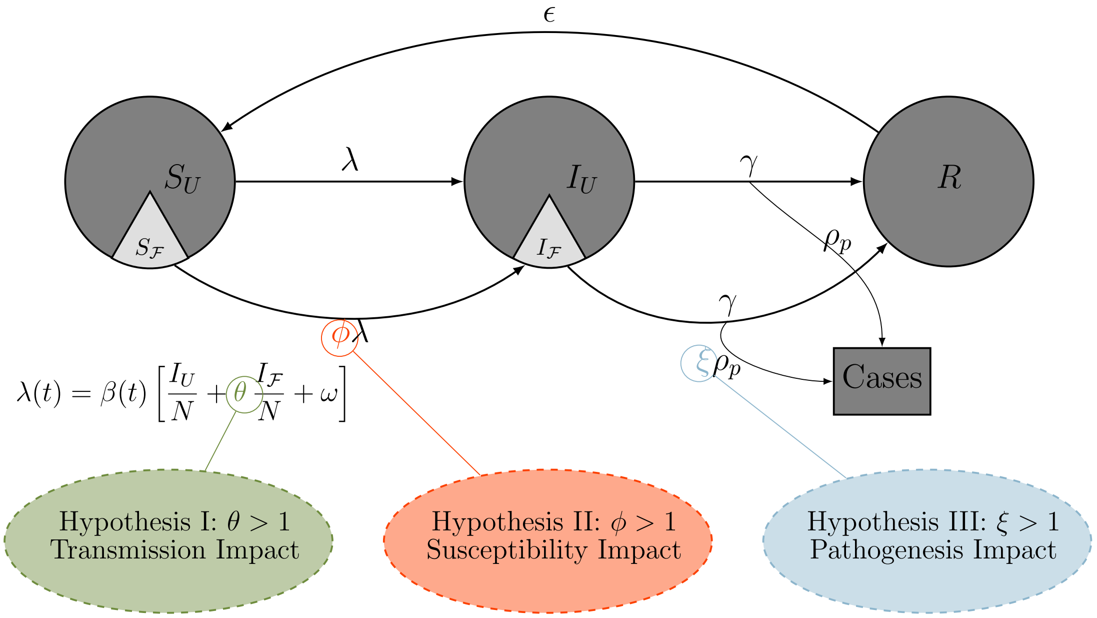

Figure S-3: Schematic representation of the model and the three hypotheses. We used a previously developed model [2] for transmission dynamics of bacterial pneumonia. In this model, the population was categorized into susceptible (S), infectious (I), and recovered (R) classes. Individuals progress along $S \to I \to R \to S$ at *per capita* rates λ, γ and ϵ, respectively. Progression of individuals recently infected with influenza were tracked separately, via classes $S_{\mathcal{F}}$ and $I_{\mathcal{F}}$. Pneumonia case reports were taken to be fraction of the infecteds as they recover. Births and deaths were present in the model, but omitted in this illustration for clarity. We test three hypothesized pathways of influenza-bacterial pneumococcal interaction: Hypothesis 1 ($\theta > 1$): Individuals infected with bacterial pneumonia, contribute more to transmission of pneumonia if they have been recently infected with influenza. Hypothesis 2 ($\phi > 1$): Individuals recently infected with influenza are more susceptible to bacterial pneumonia. Hypothesis 3 ($\xi > 1$): Individuals infected with bacterial pneumonia are more likely to be reported, if recently infected with influenza.

each: susceptibles and infecteds that are currently or very recently infected with influenza, $S_{\mathcal{F}}(t)$ and $I_{\mathcal{F}}(t)$, respectively, and the ones that are not, $S_U(t)$, and $I_U(t)$, respectively. Let $\mathcal{F}(t)$ be the number of influenza cases reported at time t. Let $\rho_{\mathcal{F}}$ be the reporting rate of influenza, ie, $\frac{\mathcal{F}(t)}{\rho_{\mathcal{F}}}$ is the number of influenza infecteds. Assuming that influenza operates independent of pneumonia, it is equally likely to be prevalent among individuals infected, susceptible and recovered with respect to pneumonia, $S_{\mathcal{F}}(t) = \dfrac{\mathcal{F}(t)}{\rho_{\mathcal{F}}\, N(t)}\, S(t)$ is the expected number of susceptibles who are also infected with influenza. Here, $N(t)$ is the population size. And, the remaining susceptibles,

$$S_U(t) = S(t) - S_{\mathcal{F}}(t) = S(t) - \frac{\mathcal{F}(t)}{\rho_{\mathcal{F}}\, N(t)}\, S(t),$$

are currently not infected with influenza.

The force of infection, or the pneumonia hazard, is composed of two parts: a part that is proportional to the fraction of infecteds at current time, and a part that is independent of this dynamics. This is slightly different from the standard formulation, which only includes the former. The addition of the latter is to account for high levels of asymptomatic carriage of the bacteria generally seen in the population [5, 6, 7]. We will also model the transmission to be seasonal. Seasonality is implemented using 6 cubic spline functions. Hence, if $I(t)$ is the number of pneumonia infecteds, and $\beta(t)$ is the seasonal transmission rate, then the force of infection is $\lambda(t) = \beta(t)\,\left[\dfrac{I(t)}{N(t)} + \omega\right]$, where ω is a constant.

We allow for the susceptibility to pneumonia among those that are infected with influenza to be different from those that are not infected with influenza. This is modeled by hazard multiplier ϕ—individuals infected with influenza experience pneumonia hazard ϕ times those that are not infected with influenza. This allows for testing hypothesis 2 ($\phi > 1$). Setting $\phi = 1$ assumes that both groups are equally susceptible, and is the null expectation for hypothesis 2.

We also allow for influenza to affect the transmission of bacterial pneumonia. We differentiate the contribution of an infected host to the hazard depending on whether or not she is infected with influenza. We introduce transmission multiplier θ—individuals recently infected with influenza contribute θ times to transmission compared to those that are not. This allows for testing hypothesis 1 ($\theta > 1$). Setting $\theta = 1$, assumes that both groups contribute equally to the hazard, and is the null expectation for hypothesis 1. To accommodate this addition, we consider the force of infection in an expanded form: $\lambda(t) = \beta(t)\,\left[\dfrac{I_U(t)}{N(t)} + \theta\,\dfrac{I_{\mathcal{F}}(t)}{N(t)} + \omega\right]$. With *per capita* recovery rate γ, loss of immunity rate ϵ, and birth/mortality rate μ, we have the following set of equations for the deterministic framework of the pneumonia model.

$$\begin{aligned}
\frac{\mathrm{d}S(t)}{\mathrm{d}t} &= \mu(N(t) - S(t)) - \lambda\, S_U(t) - \phi\,\lambda\, S_{\mathcal{F}}(t) + \epsilon\, R(t)\\
\frac{\mathrm{d}I(t)}{\mathrm{d}t} &= \lambda(t)\, S_U(t) + \phi\,\lambda(t)\, S_{\mathcal{F}}(t) - \gamma\, I(t) - \mu\, I(t)\\
\frac{\mathrm{d}R(t)}{\mathrm{d}t} &= \gamma\, I(t) - \mu\, R(t) - \epsilon\, R(t)\\
S_{\mathcal{F}}(t) &= \frac{\mathcal{F}(t)}{\rho_{\mathcal{F}}\, N(t)}\, S(t)\\
S_U(t) &= S(t) - S_{\mathcal{F}}(t) = S(t) - \frac{\mathcal{F}(t)}{\rho_{\mathcal{F}}\, N(t)}\, S(t)\\
\frac{\mathrm{d}I_{\mathcal{F}}(t)}{\mathrm{d}t} &= \phi\,\lambda(t)\, S_{\mathcal{F}}(t) - \gamma\, I_{\mathcal{F}}(t) - \mu\, I_{\mathcal{F}}(t)\\
\lambda(t) &= \beta(t)\,\left[\frac{I_U(t)}{N(t)} + \theta\,\frac{I_{\mathcal{F}}(t)}{N(t)} + \omega\right]
\end{aligned}$$

The observed variable in this model is number of pneumonia cases, C_P, observed at weekly intervals. They are assumed to be on average a fraction, ρ_p, of all infecteds in a given week as they recover. We allow for influenza to affect the reporting of pneumonia cases, via affecting the severity of the disease. We introduce severity multiplier, ξ—individuals recently infected with influenza are reported ξ times compared to those that are not. This allows for testing hypothesis 3 ($\xi > 1$). Again, setting $\xi = 1$, assumes that both groups are reported equally, and is the null expectation for hypothesis 3.

S-2.1 Incorporation of demography.

For analyses in dataset 2, we did not consider the change in populations over the 4 year period. The population sizes in the 5 major cities were held constant to the census recordings for the cities in 1920. The sizes are tabulated in Fig. 3 in the main text. For analyses in dataset 1A, and dataset 1B, we incorporated the population data from Illinois in the model. The population levels in Illinois during this period are shown in Fig. S-1. The population data is treated as a covariate in these models, and the change in population is treated as excess birth that directly enter the susceptible class.

S-2.2 Demographic and Extra-demographic Stochasticity

We translate the deterministic transmission model, defined by the the system of ordinary differential equations given above, into a stochastic model by modeling the flux between compartments to be a random process. That is, the *per capita* rates are constant and that the fluxes out of each compartment are independent, multinomial random variables over a small time interval of duration Δt,. Extra-demographic stochasticity is modeled via a gamma-distributed multiplicative white noise, dW/dt, in the transmission process [8, 9]. The standard deviation of this noise, β_{sd} is also fit along with the parameters. Hence, the force of infection is given by:

$$\lambda(t) = \beta(t) \left[\frac{I_U(t)}{N(t)} + \theta \frac{I_{\mathcal{F}}(t)}{N(t)} + \omega \right] \frac{dW}{dt}.$$

S-2.3 Measurement model

Let $H(t)$ to be the total number of new recoveries in week t, of which $H_{\mathcal{F}}(t)$ are recoveries coinfected with influenza, and the remaining $H_U(t)$ are not. We assume that the weekly case reports, C_P, are normally distributed. ie,

$$C_P \sim \mathcal{N}(\rho_p H, \sigma),$$

where, ρ_p is the reporting ratio, and σ is the standard deviation. We assume that the variance scales linearly with the mean. i.e. $\sigma^2 = c^2 \rho_p C_P$. The scalar, c, is also fit along with other parameters.

To allow for differences in reporting in pneumonia due to presence of influenza, we assume that the cases with influenza are ξ times more likely to be reported compared to the cases without influenza. This results in the following expansion of the above equation:

$$C_P \sim \mathcal{N}(\rho_p (H_{\mathcal{F}} + \xi H_U), \sigma),$$

S-3 Likelihood inference framework

We utilize the freely available software package pomp[10] that implements a partially observed Markov processes [11, 12, 13] framework to carry out likelihood based inferences. This framework consists of following four components:
(i) **Data.** The data consists of weekly case reports pneumonia.
(ii) **Covariates.** We use weekly case reports of influenza as covariates for all three datasets. For datasets 2 &

3, we additionally use annual population sizes as a covariate.
(iii) **The process model.** The process model is proposed to describe the underlying epidemiological and demographic processes (described in subsection S-2.2).
(iv) **The measurement model.** The measurement model is proposed to describe the process by which the data are reported (described in subsection S-2.3).

Consider a data set Y, consisting of observations of $y(t_j), j = 1, \ldots, n$ at n points in time. For likelihood-based inference, we calculate the likelihood that a chosen parameter set θ explains the complete data (within the confines of the process and observation models). This likelihood function $\mathcal{L}(\theta)$ is a product of conditional likelihoods, $\mathcal{L}_{t_j}(\theta)$, calculated at each time t_j for all n data points in time. If $f(y \,|\, t, \theta)$ is the probability of observing the data $y(t)$ at time t, given parameters θ (measurement model), then the likelihood and log-likelihood functions are defined as follows:

$$\begin{aligned}\mathcal{L}(\theta) &= f(y(t_1), y(t_2), \ldots, y(t_n)|\theta)\\ &= \prod_{j=1}^{n} f_\theta(y(t_j)|y(t_{j-1}), y(t_{j-2}), \ldots, y(t_1))\\ &= \prod_{j=1}^{n} \mathcal{L}_{t_j}(\theta).\\ \log \mathcal{L}(\theta) &= \sum_{j=1}^{n} \log \mathcal{L}_{t_j}(\theta).\end{aligned}$$

The Markov property of the model allows one to calculate the conditional likelihood $\mathcal{L}_{t_j}(\theta)$ sequentially starting from $t_j = t_1$. For each t_j, the likelihood function is:

$$\begin{aligned}\mathcal{L}_{t_j}(\theta) &= f_\theta(y(t_j)|y(t_{1:j-1}))\\ &= f_\theta(y(t_j)|y(t_{j-1}))\\ &= \int\int \overbrace{f_\theta(y(t_j)|x(t_j))}^{\text{observation model}} \overbrace{f_\theta(x(t_j)|x(t_{j-1}))}^{\text{process model}} \overbrace{f_\theta(x(t_{j-1})|y(t_{1:j-1}))}^{\text{filtering distribution}} \,\mathrm{d}x(t_j)\,\mathrm{d}x(t_{j-1})\end{aligned}$$

Here, $x(t_j)$ represents the state of the complete system at time t_j. Furthermore, the calculation of the filtering distribution is simplified identifying a recursive relation by applying Bayes' Theorem.

$$\begin{aligned}f_\theta(x(t_{j-1})|y(t_{1:j-1})) &= \frac{f_\theta(y(t_{j-1})|x(t_{j-1}))\, f_\theta(x(t_{j-1})|y(t_{1:j-2}))}{\int f_\theta(y(t_{j-1})|x(t_{j-1}))\, f_\theta(x(t_{j-1})|t(t_{1:j-2}))\,\mathrm{d}x(t_{j-1})}\\ &= \frac{\overbrace{f_\theta(y(t_{j-1})|x(t_{j-1}))}^{\text{observation model}}\, \overbrace{f_\theta(x(t_{j-1})|y(t_{1:j-2}))}^{\text{filtering dist.}}}{\underbrace{\mathcal{L}_{t_{j-1}}(\theta)}_{\text{cond. loglik}}}\end{aligned}$$

The density functions $f_\theta(\cdot|\cdot)$ are calculated via Sequential Monte Carlo particle filtering method [12, 14].

The likelihood functions are optimized using `mif` algorithm, which is also implemented in `pomp`[10] package. For details pertaining to the methods and implementation of the algorithm please refer to [11, 13, 10]. The range of algorithm parameters used in the inference work are shown in Table S-1.

Influenza attributable etiological fraction of pneumonia cases

Influenza attributable etiological fraction of pneumonia cases in any time interval, is taken to be the ratio of pneumonia cases as a result of influenza to the total pneumonia cases in the given time interval. Let $H(t)$

Algorithm parameter	Description	Range
Np	Number of particles	5000 - 50000
Nmif	Number of mif iterations	50 - 150
var.factor	Starting particle distribution	2 - 3
cooling.factor	Exponential cooling factor	0.95 - 0.99
ic.lag	Fixed-lag smoothing of initial-value parameters	2 - 4 years
rw.sd	Intensity of the random walk	0.005 - 0.02

Table S-1: Parameters used in inference algorithm with their ranges.

be the total number of new recoveries in week t, of which $H_{\mathcal{F}}(t)$ are recoveries coinfected with influenza. With the normal reporting process, with the reporting ratio, ρ_p, and the standard deviation σ, the total reported cases are $C_P \sim \mathcal{N}(\rho_p H_{\mathcal{F}}, \sigma)$, and total reported cases of pneumonia infections with influenza are $C_P^{\mathcal{F}} \sim \mathcal{N}(\xi \rho_p H_{\mathcal{F}}, \sigma)$. Recall, ξ is the hypothesized measure of severity impact. The influenza attributable etiological fraction of pneumonia cases during week t, $E_{\mathcal{F}}(t)$ is given by:

$$E_{\mathcal{F}}(t) = \frac{C_P^{\mathcal{F}}(t)}{C_P(t)}.$$

We estimate C_P, and $C_P^{\mathcal{F}}$, using separate MLE models for datasets 1A, 1B, and 2, which are presented in table S-2. Note that $\xi = 1$ in these MLEs. We use 1000 simulations of the MLE models to find the mean and the 95% confidence intervals for these estimates.

S-4 Additional Results

S-4.1 General features of pneumonia epidemiology

In Table S-2, we list all the parameters used in the pneumonia model, including the ones that are estimated. We estimated 16 parameters altogether: 2 for initial conditions, 6 for the shape of the seasonality, 3 for reporting processes, 3 for epidemiology of bacterial pneumonia, 1 for interaction, 1 for extra-demographic stochasticity.

In Table S-2, we also show the estimates of all other parameters corresponding to the MLE. The simulations arising from the MLE model, in comparison to the null model (with no interaction) for Dataset I are shown in Fig. S-7. Pneumonia is highly seasonal, seen in the difference of β_is—the transmission during the high season is about 7 times that during the low season. The infection is estimated to last a little less than a week (~ 6 days). The immunity imparted by the infection is estimated to last long (~ 20 years), although, this estimate is likely to not be robust since we only used 4 years of data. The symptomatic ratio of pneumonia is estimated to be about 3%.

A defining characteristic of the epidemiology of pneumonia is the large carriage rate. It is believed that a substantial fraction of the population can be carrying pneumococcal bacteria in the naso-pharynx without showing any symptom [15, 16]. The duration of carriage can vary between weeks to months. Yet, it is unclear how the carriage affects invasive pneumonia [17, 15, 18]. Here, we estimate the contribution of carriage to the total force of infection, ω, in Dataset I. In Fig. S-4, we show the likelihood profile of this parameter ω. We estimate this to be between 0.047 to 0.234. This suggests that pneumonia epidemiology is primarily driven by asymptomatic carriage.

S-4.2 Timescale of the interaction

In the original model, we hypothesized that individuals currently or very recently infected (up to a week) with influenza face different hazard rate compared to those that do not. Here, we hypothesize that individuals infected up to three weeks in the past with influenza face different hazard rates. Let $\mathcal{F}_1(t)$, and $\mathcal{F}_2(t)$ respectively be the influenza case reports 1 week and 2 weeks in the past, i.e. $\mathcal{F}_1(t) = \mathcal{F}(t-1)$, and $\mathcal{F}_2(t) = \mathcal{F}(t-2)$. Let $S_{\mathcal{F}_1}$, and $S_{\mathcal{F}_2}$ respectively be the sizes of the susceptible population that were infected with influenza between 1-2 weeks, and 2-3 weeks in the past. We estimate these quantities in the same way we estimated $S_{\mathcal{F}}$. i.e., $S_{\mathcal{F}_1} = \dfrac{\mathcal{F}_1(t)}{\rho_{\mathcal{F}}\, N}\, S$, and $S_{\mathcal{F}_2} = \dfrac{\mathcal{F}_2(t)}{\rho_{\mathcal{F}}\, N}\, S$. With $S_{\mathcal{F}}$, defined the same way as in the original model, and S_U being the rest of the susceptible population, the susceptible compartment is now divided into 4 sub-compartments: $S_{\mathcal{F}}$, $S_{\mathcal{F}_1}$, $S_{\mathcal{F}_2}$, and S_U. Let the hazard rates faced by each of the susceptible sub-compartments be $\phi\,\lambda$, $\phi_1\,\lambda$, $\phi_2\,\lambda$, and λ, respectively.

Parameter	Description	Fit	MLE (Estimated Range†)		
			**Dataset 2 (NYC)**	**Dataset 1A**	**Dataset 1B**
N	Population size	fixed/ covariate	fixed at 5,620,048	covariate	covariate
μ	Host birth/mortality rate (year^{-1})	fixed	0.02	0.02	0.02
γ	Host recovery rate (year^{-1})	yes	60.92 $(52.69 - 68.15)$	24.92 $(18.59 - 37.87)$	28.75 $(25.77 - 36.49)$
ϵ	Loss of immunity rate (year^{-1})	yes	0.04 $(0.04 - 0.10)$	0.95 $(0.23 - 4.4)$	0.10 $(0.10 - 0.54)$
$\rho_{\mathcal{F}}$	Symptomatic ratio (influenza)	yes	0.032 $(0.01 - 0.07)$	0.095 $(0.02 - 0.29)$	0.09 $(0.04 - 0.13)$
ρ_P	Symptomatic ratio (pneumonia)	yes	0.056 $(0.03 - 0.06)$	0.026 $(0.01 - 0.13)$	0.13 $(0.09 - 0.18)$
ω	Carriage-dependent hazard	yes	0.095 $(0.04 - 0.15)$	0.016 $(0.004 - 0.05)$	0.0086 $(0.007 - 0.009)$
β_1	Transmission rate (1st seas. basis) (year^{-1})	yes	15.68 $(12.01 - 34.53)$	19.07 $(8.71 - 28.64)$	17.57 $(7.13 - 17.57)$
β_2	Transmission rate (2nd seas. basis) (year^{-1})	yes	22.12 $(17.48 - 55.81)$	16.09 $(6.47 - 25.82)$	16.54 $(6.73 - 16.54)$
β_3	Transmission rate (3rd seas. basis) (year^{-1})	yes	19.78 $(15.78 - 48.16)$	15.28 $(6.50 - 23.90)$	14.10 $(5.96 - 14.41)$
β_4	Transmission rate (4th seas. basis) (year^{-1})	yes	3.09 $(2.47 - 8.41)$	10.21 $(3.72 - 16.72)$	10.25 $(4.21 - 10.25)$
β_5	Transmission rate (5th seas. basis) (year^{-1})	yes	4.25 $(3.26 - 9.57)$	13.67 $(5.45 - 21.34)$	13.79 $(5.49 - 13.79)$
β_6	Transmission rate (6th seas. basis) (year^{-1})	yes	8.38 $(6.25 - 20.48)$	15.48 $(5.92 - 23.66)$	13.72 $(5.87 - 13.72)$
β_{sd}	Std. deviation of extra-demographic noise	yes	0.046 $(0.043 - 0.050)$	0.027 $(0.021 - 0.034)$	0.022 $(0.016 - 0.022)$
c	Observation model scalar	yes	0.97 $(0.74 - 1.24)$	1.14 $(0.91 - 1.26)$	0.53 $(0.53 - 0.97)$
ϕ	Enhancement	yes	80 $(25 - 150)$	100 $(1 - 300)$	110 $(65 - 200)$
$S(0)$	Initial susceptible fraction	yes	0.08 $(0.08 - 0.15)$	0.64 $(0.55 - 0.77)$	0.34 $(0.34 - 0.69)$
$I(0)$	Initial infectious fraction	yes	1.15×10^{-5} $(1.1 - 3.6 \times 10^{-5})$	7.47×10^{-3} $(2.5 - 8.9 \times 10^{-3})$	2.7×10^{-3} $(2.7 - 4.0 \times 10^{-3})$
$R(0)$	Initial recovered fraction	fixed	0.92 $(0.85 - 0.92)$	0.36 $(0.22 - 0.45)$	0.66 $(0.30 - 0.66)$

Table S-2: Model parameters and their estimated range for epidemiology of pneumonia. † Estimated range for each parameter shown in this table corresponds to the respective range observed while calculating 95% confidence interval for ϕ. Note that the ranges presented here are *not* necessarily 95% confidence intervals for each parameter, except for parameter ϕ, for which the 95% confidence interval was calculated.

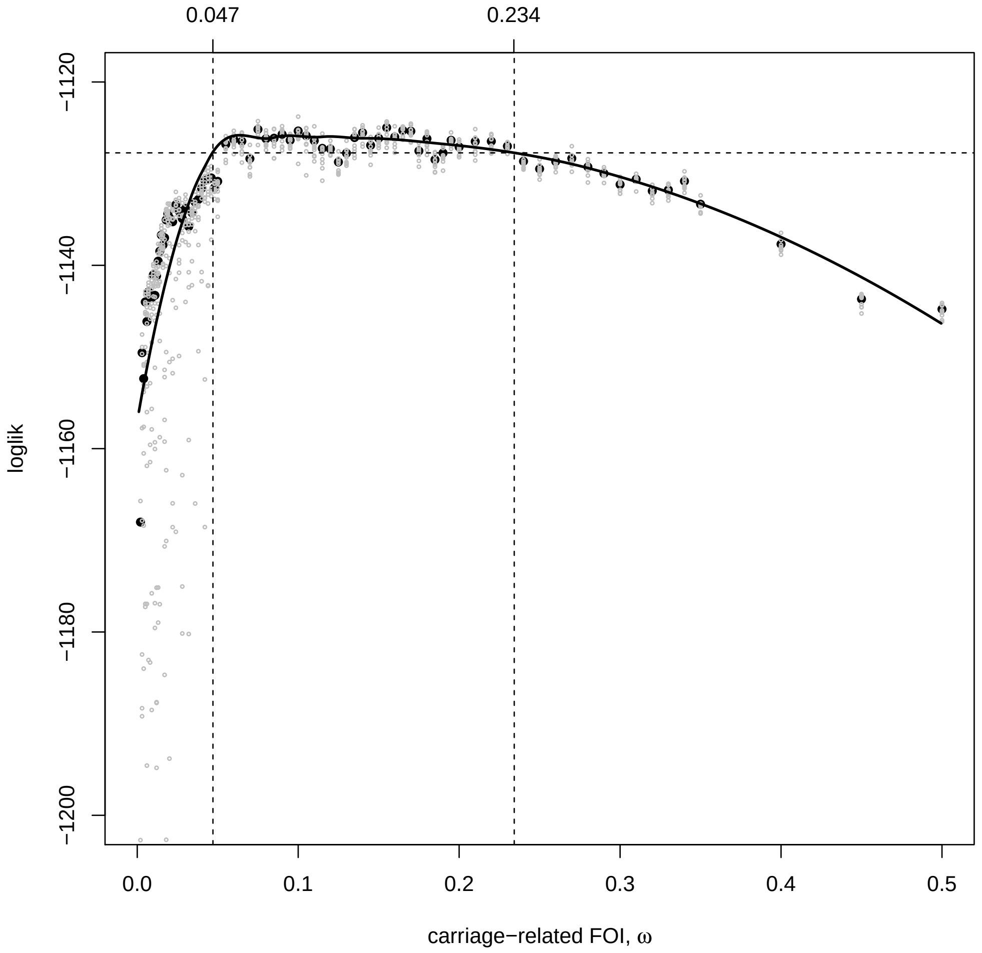

Figure S-4: Estimate of carriage related hazard (ω) using likelihood profile. Likelihood for each profile point (filled black circle) is mean of the likelihoods in 10 replication (shown as open gray circles). All likelihoods are presented in natural log scale as log-likelihoods. The values of ω that fall between the two vertical dashed lines are within the 95% confidence interval. This estimation was based dataset 2 (New York City).

The complete lagged-interaction model is described by the following set of equations:

$$
\begin{aligned}
\frac{\mathrm{d}S}{\mathrm{d}t} &= \mu(N-S) - \lambda\, S_U - \phi\,\lambda\, S_{\mathcal{F}} - \phi_1\,\lambda\, S_{\mathcal{F}_1} - \phi_2\,\lambda\, S_{\mathcal{F}_2} + \epsilon\, R \\
\frac{\mathrm{d}I}{\mathrm{d}t} &= \lambda\, S_U + \phi\,\lambda\, S_{\mathcal{F}} + \phi_1\,\lambda\, S_{\mathcal{F}_1} + \phi_2\,\lambda\, S_{\mathcal{F}_2} - \gamma\, I - \mu\, I \\
\frac{\mathrm{d}R}{\mathrm{d}t} &= \gamma\, I - \mu\, R - \epsilon\, R \\
S_{\mathcal{F}} &= \frac{\mathcal{F}(t)}{\rho_{\mathcal{F}}\, N}\, S \\
S_{\mathcal{F}_1} &= \frac{\mathcal{F}_1(t)}{\rho_{\mathcal{F}}\, N}\, S \\
S_{\mathcal{F}_2} &= \frac{\mathcal{F}_2(t)}{\rho_{\mathcal{F}}\, N}\, S \\
S_U &= S - S_{\mathcal{F}} - S_{\mathcal{F}_1} - S_{\mathcal{F}_2} = S - \frac{\mathcal{F}(t)}{\rho_{\mathcal{F}}\, N}\, S - \frac{\mathcal{F}_1(t)}{\rho_{\mathcal{F}}\, N}\, S - \frac{\mathcal{F}_2(t)}{\rho_{\mathcal{F}}\, N}\, S
\end{aligned}
$$

Our estimates for ϕ_1 and ϕ_2, for pneumonia are shown in Fig. S-5. We find that the null models, $\phi_1 = 1$ and $\phi_2 = 1$ are within the 95% confidence interval, indicating that there is no evidence of susceptibility enhancement among cohorts that were infected with influenza more than 1 weeks prior.

S-4.3 The three hypotheses

Here, we explore the three different pathways of interaction in dataset 2, New York City in further detail. In particular, we are interested in understanding the tradeoffs between the each of the hypotheses. To do so, we look at likelihood surfaces, when profiled over two of the three parameters holding other to the null. Likelihood surfaces over ϕ-ξ plane (Fig. S-6B), ϕ-θ plane (Fig. S-6E) and θ-ξ plane (Fig. S-6H), show how two parameters are associated. When the model is allowed to account susceptibility impact (panels B and E), the maximum likelihood models select for only susceptibility impact. When the model is not allowed to account for susceptibility impact (panel H), the maximum likelihood model selects for pathogenesis impact (over transmission). But, the MLE value in this instance is significantly smaller that MLE values obtained in the instances where the model is allowed to account for susceptibility impact.

S-4.4 Out-of-fit Predictions in other cities

We test the ability of the models we formulated on the basis of New York data (Dataset II) to predict pneumonia cases in other major cities during the same time period (see Fig. S-2). Using (i) parameter values corresponding to the MLE (S-2) and the null model from the New York data, and (ii) population and influenza data from the respective cities, we simulate the model forward and compare the predictions to the pneumonia case data in these cities. Simulations of the out-of-fit predictions based on both of the models are shown in Fig. S-8. The MLE model shows some ability to capture inter-annual variability in the data, that the null model cannot.

S-4.5 Fraction of pneumonia cases attributable to influenza.

We calculated the fraction of pneumonia cases that are attributable to influenza in datasets 2 (New York City), dataset 1A (Illinois, before PCV), dataset 1B (Illinois, after PCV). As shown in Fig. S-9, influenza-attributable etiological fraction of pneumonia cases show variability between different datasets. In New York City, between 1920 and 1924, the fractions of pneumonia cases attributable to influenza were between 2%-7% annually. In Illinois, the fractions were relatively smaller: $<2\%$ annually in both datasets. As expected, the effect

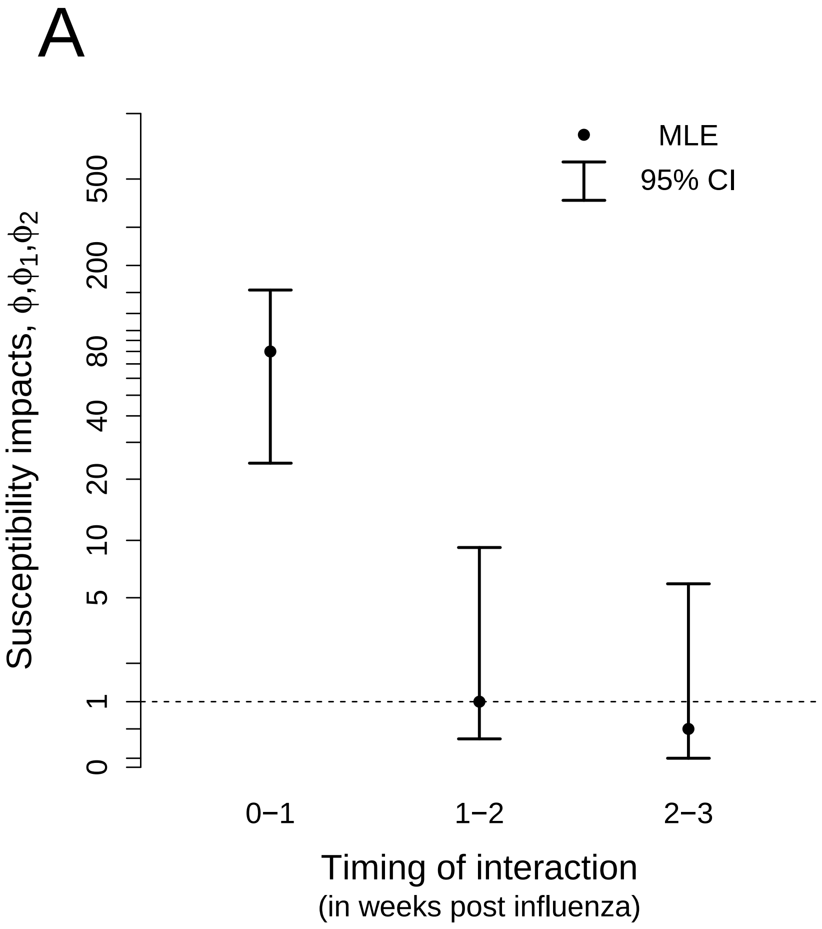

Figure S-5: Susceptibility impacts in pneumonia at different times post influenza infection. Estimates for susceptibility impacts are calculated in individuals up to 1 week post influenza, ϕ, between 1 and 2 weeks post influenza, ϕ_1, and between 2 and 3 weeks post influenza, ϕ_2. Black dots indicate the MLEs, and the vertical bars indicate the 95% confidence intervals. $\phi = 1$, $\phi_1 = 1$ and $\phi_2 = 1$ are the respective null models. This estimation was based dataset 2 (New York City).

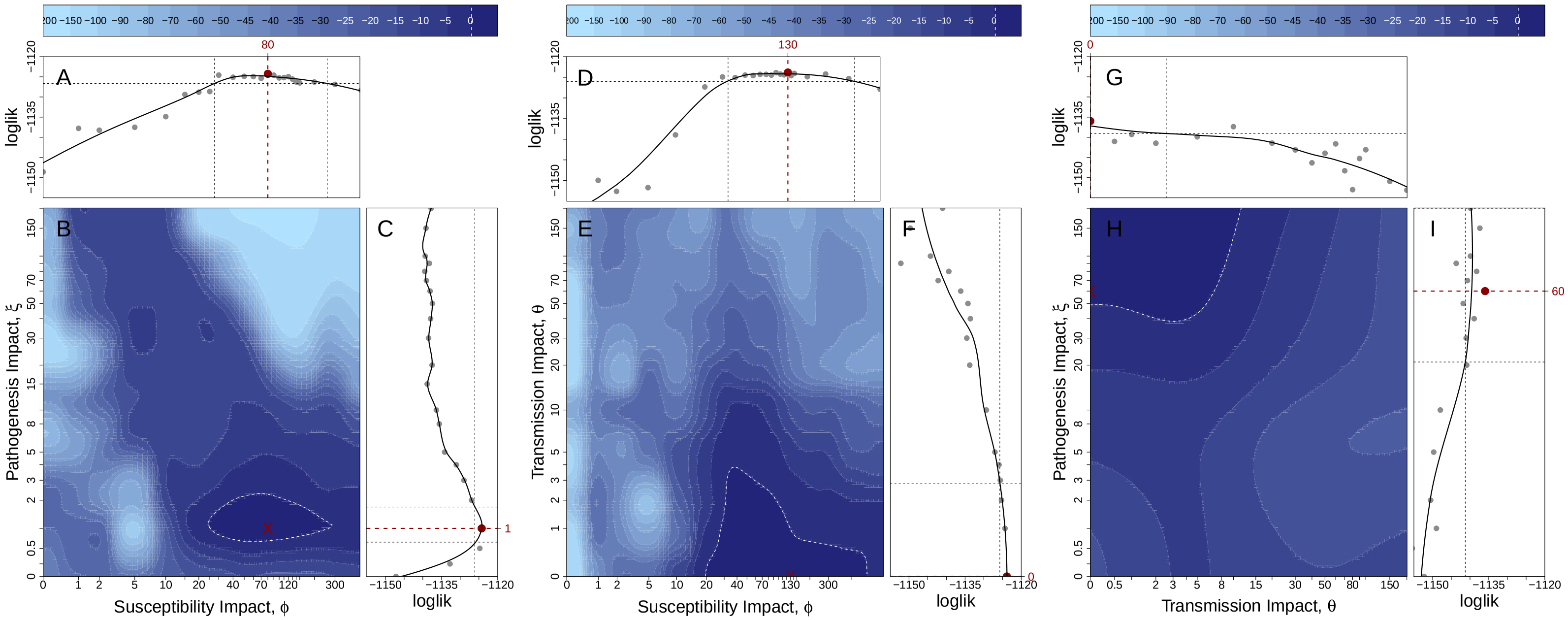

Figure S-6: Likelihood surfaces of parameters relating to the three hypotheses in New York city (dataset 2). The color-coded contours represent log-likelihood surfaces in (B) ϕ-ξ plane, with $\theta = 1$; (E) ϕ-θ plane, with $\xi = 1$; and (H) θ-ξ plane, with $\phi = 1$. A point on the surface shows the log-likelihood (relative to the 95% confidence level) for corresponding parameters that are plotted, in the absence of the parameter that is set to the null value, e.g. the color corresponding to the point (20,10) in (B) is the relative log likelihood for the model with $\phi = 20$, $\xi = 10$, and $\theta = 1$. In each surface plot, the region bounded by the dashed white line is the 95% confidence regions, and the red cross represent the MLE. Plotted on the top and right sides of each surface plot are likelihood profiles for individual parameters, which are constructed by projecting the likelihood surface on to respective axes. Note that these are different from the true likelihood profiles in that they profiles with one of the parameters set to the null value, e.g. (A) is the likelihood profile for ϕ, with $\theta = 1$. The black dotted lines show 95% confidence intervals, and the dashed red line shows the MLE.

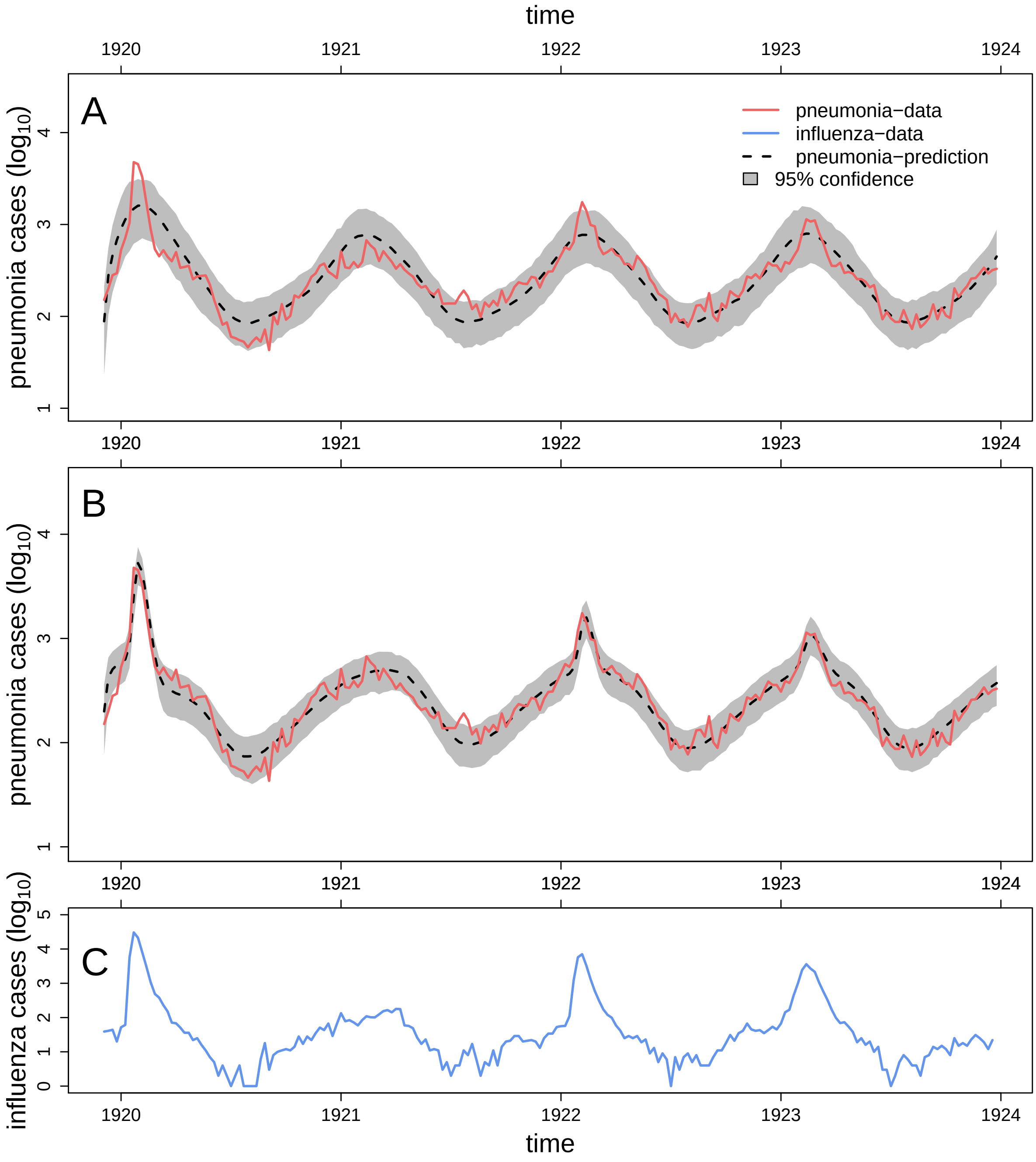

Figure S-7: Simulations from the models fit to New York city data (dataset 2). We compare the simulations from two fitted models with the data—(A) The null model, $\phi = 1$, and (B) The maximum likelihood estimate model (MLE-model), with enhancement, $\phi = 80$. Influenza cases are shown in (C). The model without the susceptibility enhancement is able to capture the seasonality in the pneumonia cases, but cannot explain the variability in the annual peaks.

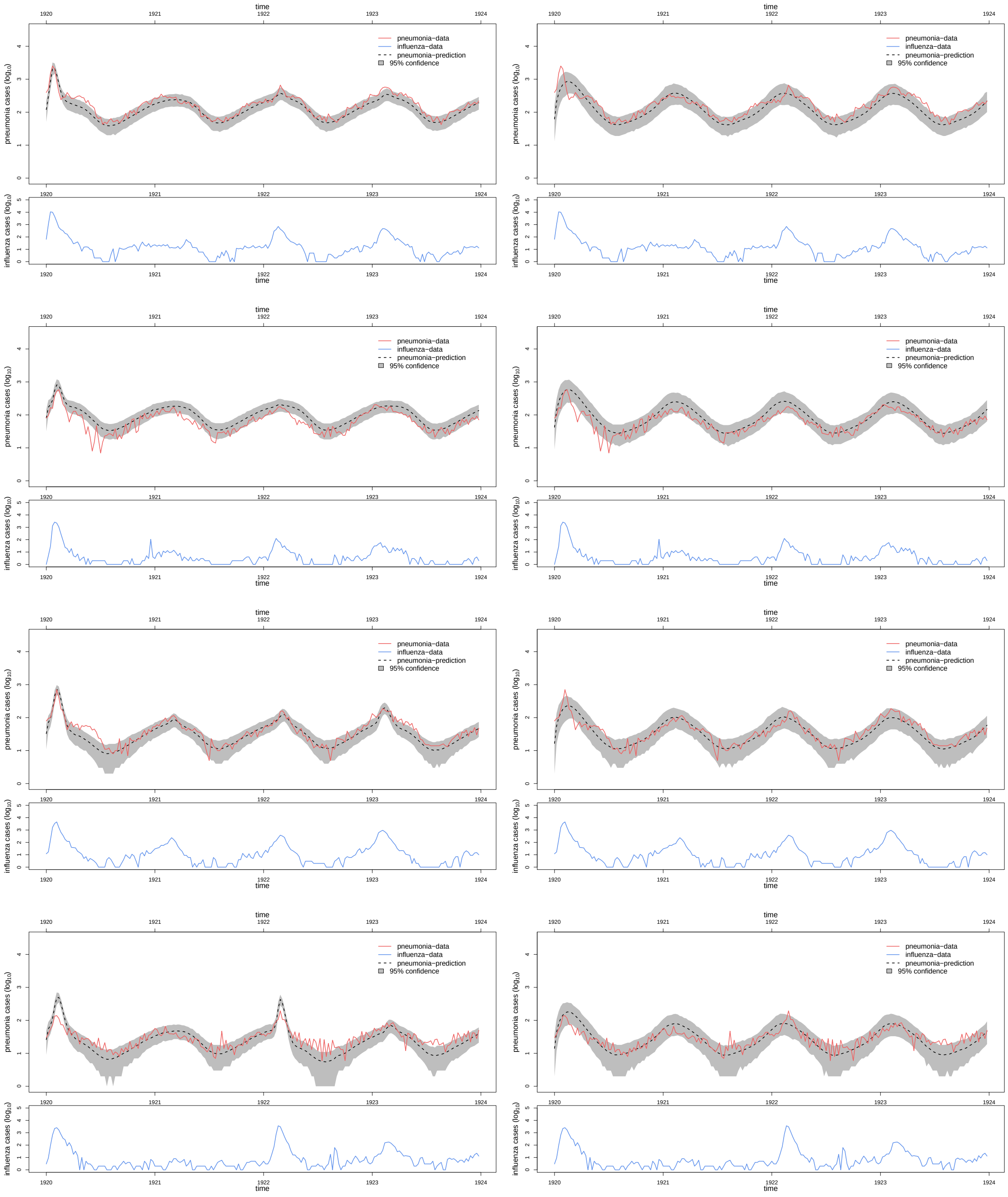

Figure S-8: Predictions for 4 other major US cities—Chicago (1st row), Philadelphia (2nd row), Baltimore (3rd row), and Los Angeles (4th row)– during the same time frame, using the MLE model (1st column), and the null model (2nd column).

of influenza was also highly seasonal, with most of effect observed the during the high influenza part of the season.

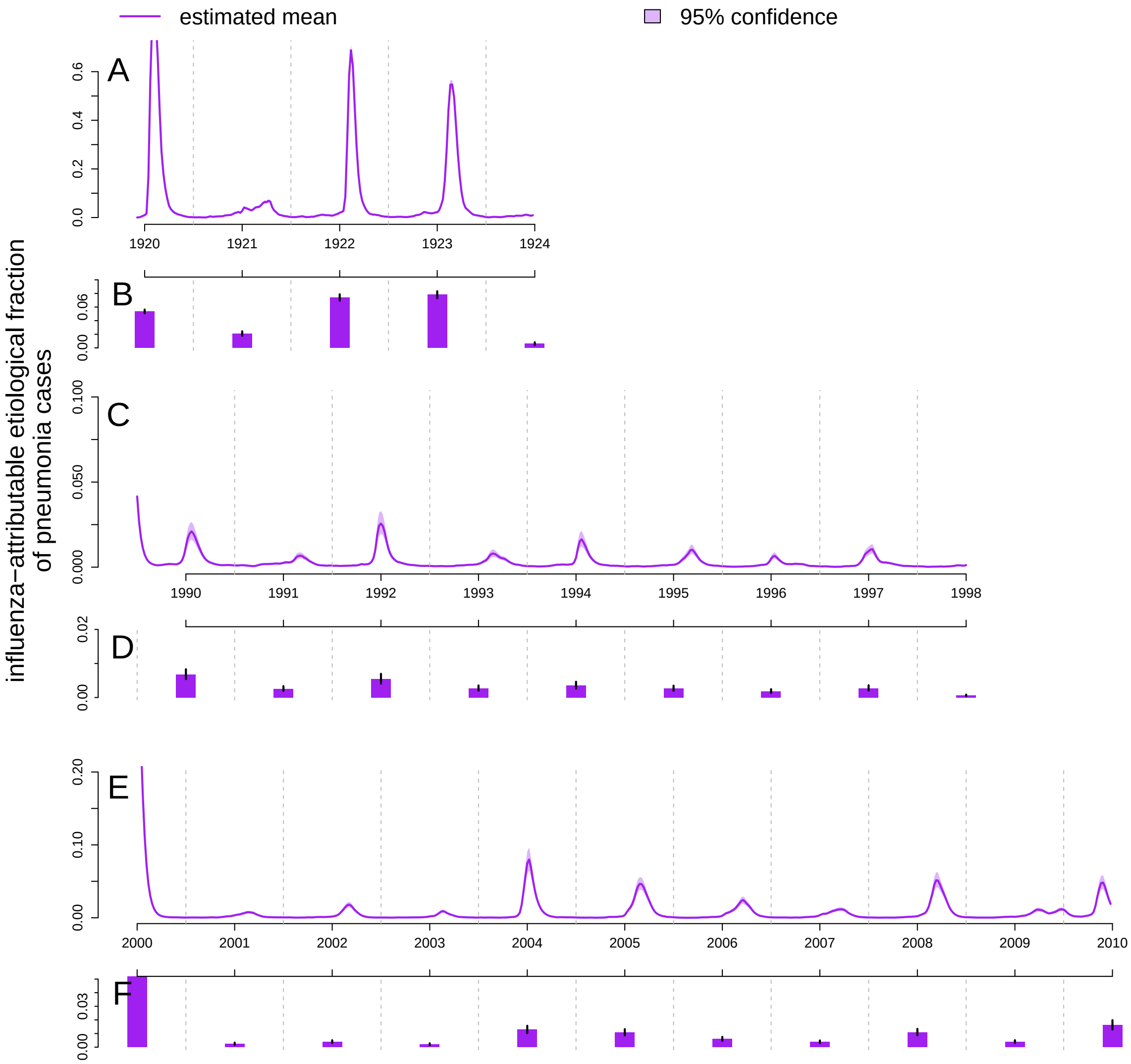

Figure S-9: Impact of influenza on the incidence of pneumonia. Based on the interaction models, we estimate the influenza-attributable etiological fraction of pneumonia cases in dataset 2 (New York City) in panels A and B, dataset 1A (Illinois, before PCV) in panels C and D, and dataset 1B (Illinois, after PCV) in panels E and F. Influenza-attributable etiological fraction of pneumonia cases in any time interval is taken to be the ratio of pneumonia cases as a result of influenza to the total pneumonia cases in the given time interval. Panels A, C and E show the weekly variations in the etiological fractions, panels B, D, and F show the fractions averaged over annual periods, midyear to midyear, for intervals shown in A, C, and E, respectively.

S-5 Sensitivity analyses

S-5.1 Susceptibility enhancement and influenza symptomatic ratio

Since we estimate the symptomatic ratio of influenza along with the enhancement, we expect the uncertainty in symptomatic ratio to affect our ability to infer enhancement. To delve further, we constructed a likelihood surface with ϕ on one axis and symptomatic ratio of influenza, $\rho_{\mathcal{F}}$ on the other, for pneumonia data in New York City (Dataset I). As seen in Fig. S-10, the peak of the likelihood surface forms a ridge, starting from ($\phi \approx 25$, $\rho_{\mathcal{F}} \approx 1.5\%$), to ($\phi \approx 140$, $\rho_{\mathcal{F}} \approx 5\%$). We estimate the symptomatic ratio to fall between 1.5% to 5%. Furthermore, the estimate of enhancement increases with this ratio. This partially explains the broad confidence interval we get for ϕ, and further suggests that accuracy in the measurement of influenza can help quantify the enhancement more accurately.

S-5.2 Decoupling seasonality

In the original model, we allowed for the seasonal force of infection to be composed of two parts; one that is proportional to the fraction of infectious in the population, and the other that is independent. A natural question is whether the two components have different seasonality, and importantly whether the inference of the enhancement is sensitive to this. In this dual-seasonality model, we vary the force of infection in the following way:

$$\lambda(t) = \beta^1_{\text{seas}} \frac{I}{N} + \beta^2_{\text{seas}}\, \omega$$

Note that setting $\beta^1_{\text{seas}} = \beta^2_{\text{seas}} = \beta_{\text{seas}}$, is equivalent to the original model. Hence the original model is nested inside this model, with this model adding 6 more parameters.

Shown in Fig. S-11 is the likelihood profile of enhancement, ϕ using this model. The likelihood profile obtained with this model remains mostly unaltered, when compared with the original model shown in the main text. The shape of the seasonality also does not change significantly. (See Fig. S-12) This suggests that the seasonality of transmission related to carriage is similar to that related to invasive pneumonia.

S-5.3 Predictions of coinfections during the 1918 pandemic.

The MLE$^+$ model, used for the prediction of incidences of pneumonia and coinfections across 34 army camps during 1918 pandemic, included changes in 3 parameters in the MLE model, namely (i) birthrate (interpreted in this scenario as turnover rate) μ, (ii) reporting ratio for influenza $\rho_{\mathcal{F}}$, and (iii) reporting ratio for pneumonia, ρ_p. Here (Fig. S-13) we examine the sensitivity of the predictions as result of changes in these three parameters. The predictions are fairly robust to moderate variations in all three parameters.

S-5.4 Simulation based predictions of pneumonia excluding pneumococcal pneumonia.

We simulated models of pneumonia (as presented in the main text) and models of pneumococcal pneumonia (as presented in [2]), to generate simulation based predictions of pneumonia excluding pneumococcal pneumonia. By subtracting predictions of pneumococcal pneumonia from predictions of pneumonia, we construct simulation based predictions of pneumonia excluding pneumococcal pneumonia. The null predictions of pneumonia excluding pneumococcal pneumonia were generated by subtracting null-model predictions of pneumococcal pneumonia from null-model predictions of pneumonia. Similarly, the MLE predictions of pneumonia excluding pneumococcal pneumonia were generated by subtracting MLE-model predictions of pneumococcal pneumonia from MLE-model predictions of pneumonia. These null and MLE predictions of pneumonia excluding pneumococcal pneumonia are compared against the data. The data for pneumonia excluding pneumococcal pneumonia is created by subtracting weekly reports of pneumococcal pneumonia from pneumonia.

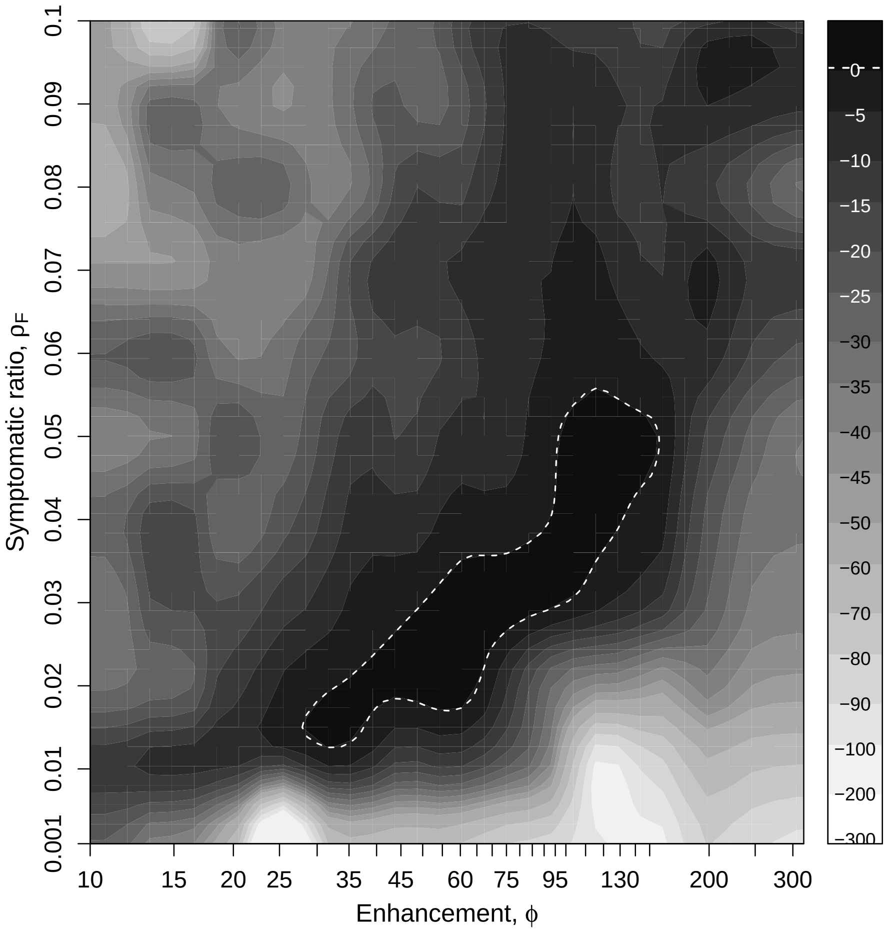

Figure S-10: Likelihood surface for enhancement, ϕ and influenza symptomatic ratio, $\rho_{\mathcal{F}}$. Plotted are log-likelihoods, with 0 set at the 95% confidence interval, shown by the dashed line. The surface is constructed by first maximizing likelihoods at various sampled points in the two-dimensional grid, each consisting a pair of ϕ and $\rho_{\mathcal{F}}$, and then fitting a smooth surface. This figure shows that the ability to infer the enhancement factor is somewhat lost by having to simultaneously infer symptomatic ratio for influenza. The lower end of the estimate for enhancement ($\phi \approx 25$) corresponds to symptomatic ratio of 1.5%, whereas the higher end of the estimate ($\phi \approx 160$) corresponds to asymptomatic ratio of 5%. This estimation was based dataset 2 (New York City).

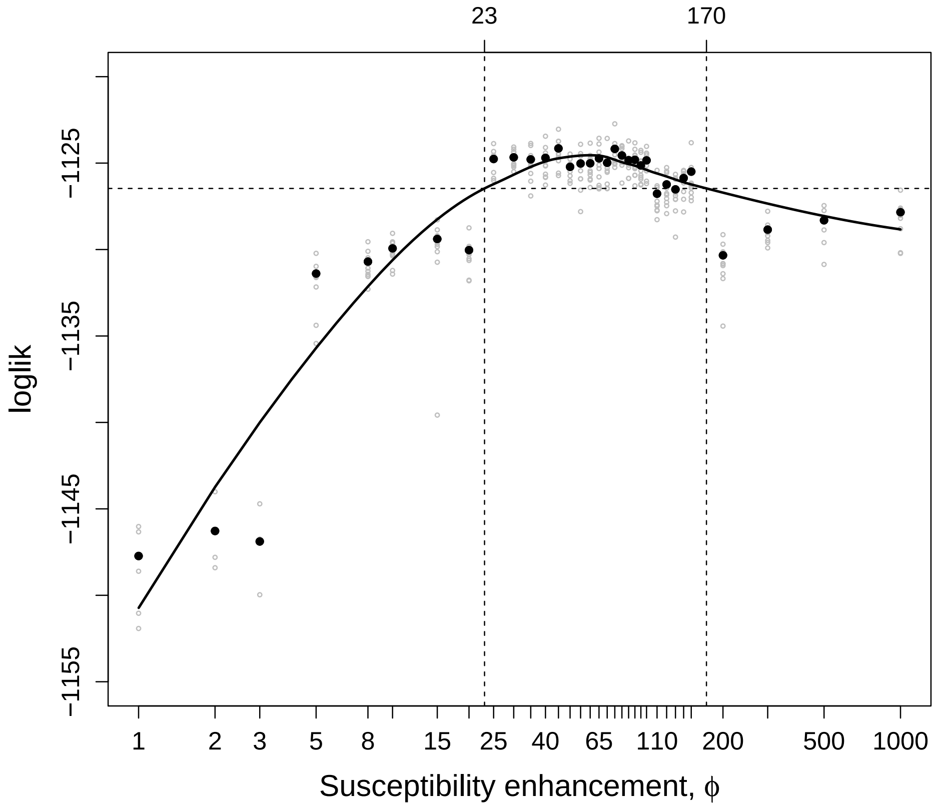

Figure S-11: Likelihood profile for enhancement, ϕ, dual-seasonality model. Likelihood for each profile point (filled black circle) is mean of the likelihoods in 10 replication (shown as open gray circles). All likelihoods are presented in natural log scale as log-likelihoods. Enhancement, ϕ, is plotted in the log_{10} scale. The values of ϕ that fall between the two vertical dashed lines are within the 95% confidence interval. This estimation was based dataset 2 (New York City).

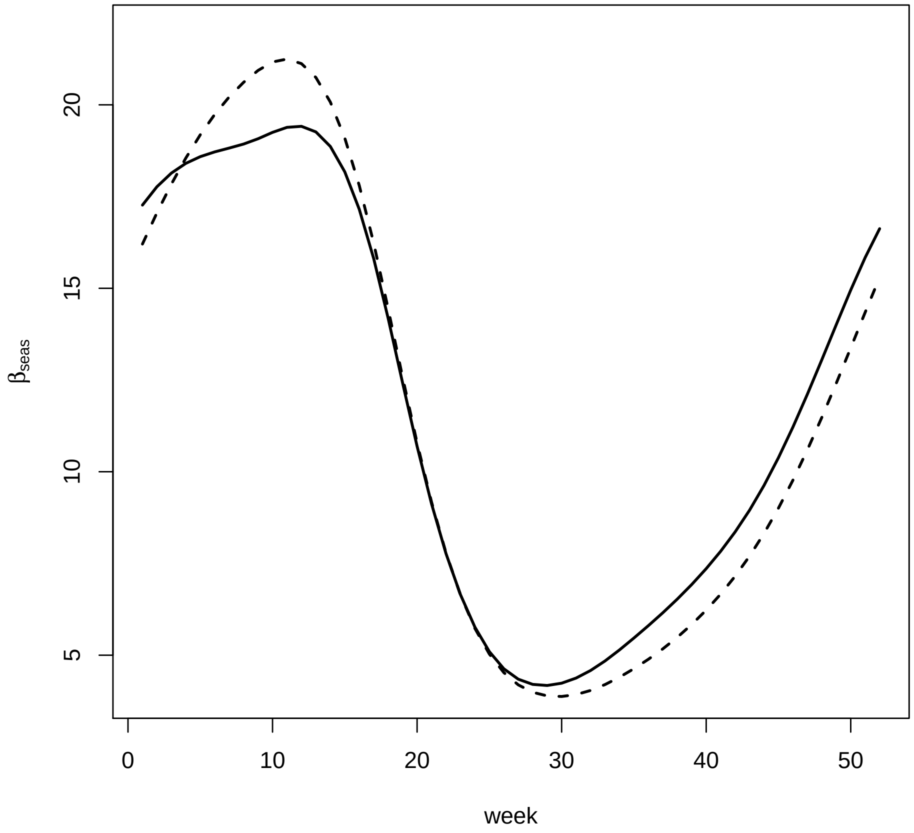

Figure S-12: Seasonality of two pathways. Shown are the seasonality in the transmission through two pathways—β^1_{seas} (solid line) and β^2_{seas} (dashed line) This estimation was based dataset 2 (New York City).

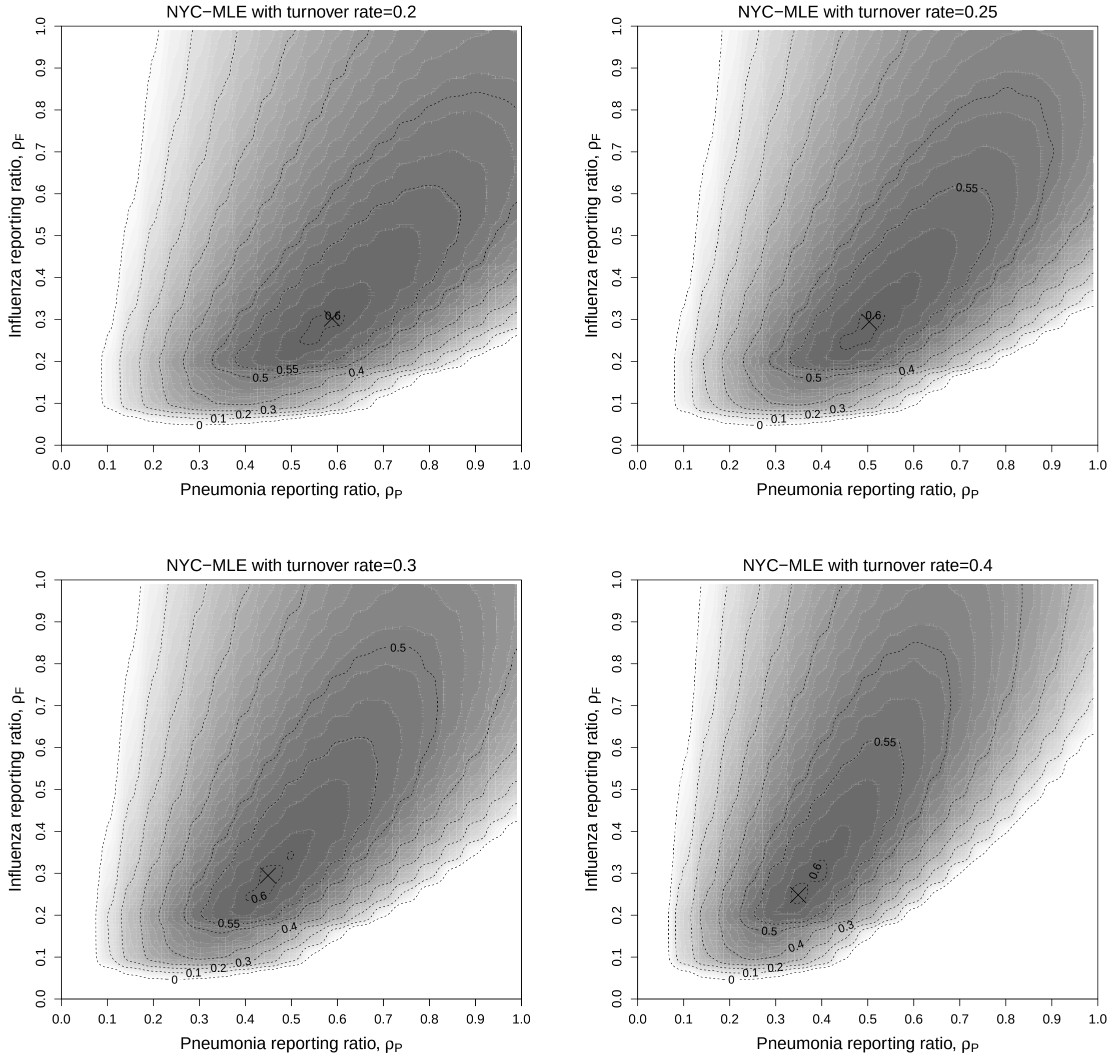

Figure S-13: Sensitivity of coinfection predictions across 34 army camps during 1918 pandemic. Plotted are contours of R^2 goodness of fit between the data and the predictions of coinfections during the three months (S,O,N) spanning the Fall wave of 1918 pandemic. The four figure panels show annual turnover rates of [Top-Left] 0.2, [Top-Right] 0.25, [Bottom-Left] 0.3 and [Bottom-Right] 0.4, and the vertical and horizontal axes in each of the figure panel shows reporting rates of influenza and pneumonia, respectively.

Shown in Fig. S-14 are comparisons for Illinois data before the PCV vaccine, and shown in Fig. S-15 are the comparisons for Illinois data after the PCV vaccine. The R^2 goodness of fit for the respective null models were 0.471 and 0.697 in pre-vaccine data and the post-vaccine data, respectively. In comparison, the goodness of fit for the respective MLE models were 0.532 and 0.745 in pre-vaccine data and the post-vaccine data, respectively, and superior to the predictions from the null models. This suggests that interactions between influenza and bacterial pneumonia are not limited to pneumococcal pneumonia, without having fully inferred the interaction between influenza and non-pneumococcal pneumonia.

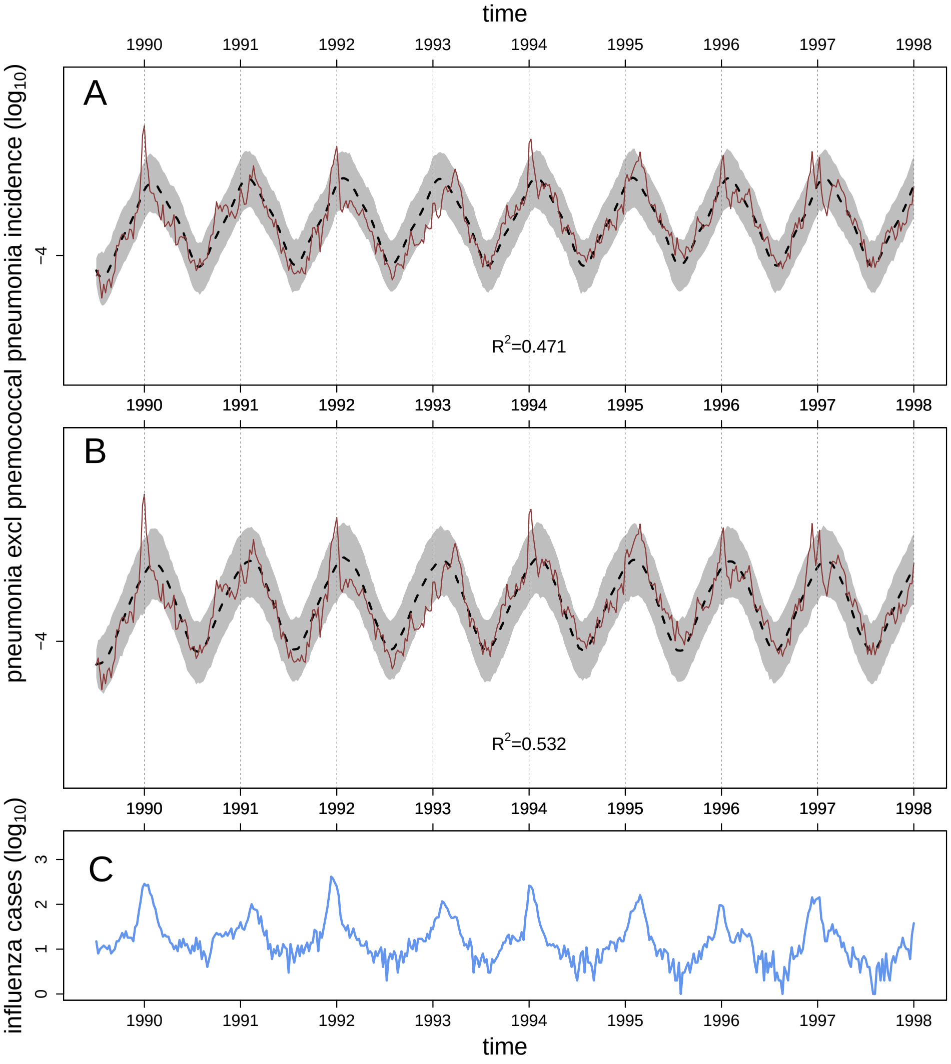

Figure S-14: Comparisons of model simulations and data for pneumonia excluding pneumococcal pneumonia in Illinois data before the introduction of PCV vaccine. [A] Simulation based predictions of the null model. [B] Simulation based predictions of the MLE model. [C] Weekly influenza hospitalization reports. Null predictions of pneumonia excluding pneumococcal pneumonia were generated by subtracting null predictions of pneumococcal pneumonia (based on the null model from Shrestha et al [2]) from null predictions of pneumonia.

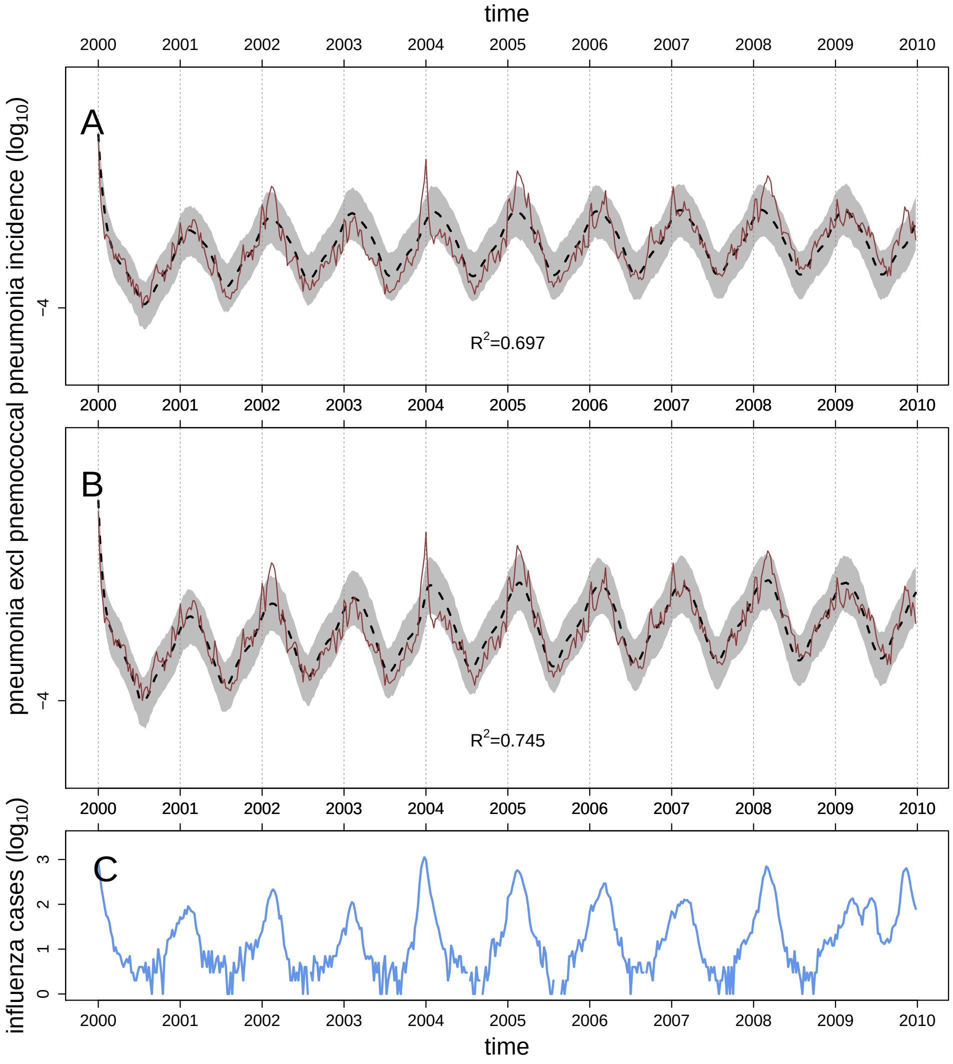

Figure S-15: Comparison of model simulations and data for pneumonia excluding pneumococcal pneumonia in Illinois data after the introduction of PCV vaccine. [A] Simulation based predictions of the null model. [B] Simulation based predictions of the MLE model. [C] Weekly influenza hospitalization reports. Null predictions of pneumonia excluding pneumococcal pneumonia were generated by subtracting null predictions of pneumococcal pneumonia (based on the null model from Shrestha et al [2]) from null predictions of pneumonia.

References

[1] van Panhuis, W. G. *et al.* Contagious diseases in the united states from 1888 to the present. *New England Journal of Medicine* **369**, 2152–2158 (2013). PMID: 24283231, arXiv:http://www.nejm.org/doi/pdf/10.1056/NEJMms1215400.

[2] Shrestha, S. *et al.* Identifying the interaction between influenza and pneumococcal pneumonia using incidence data. *Science Translational Medicine* **5**, 191ra84 (2013). arXiv:http://stm.sciencemag.org/content/5/191/191ra84.full.pdf.

[3] Anderson, R. M. & May, R. M. *Infectious Diseases of Humans; Dynamics and Control* (Oxford University Press, Oxford, 1991).

[4] Keeling, M. & Rohani, P. *Modelling Infectious Diseases* (Princeton University Press, Princeton, 2008).

[5] Granat, S. *et al.* Longitudinal study on pneumococcal carriage during the first year of life in Bangladesh. *Pediatr. Infect. Dis. J.* **26**, 319–324 (2007).

[6] Hill, P. C. *et al.* Nasopharyngeal carriage of *Streptococcus pneumoniae* in Gambian infants: A longitudinal study. *Clin. Infect. Dis.* **46**, 807–814 (2008). arXiv:http://cid.oxfordjournals.org/content/46/6/807.full.pdf+html.

[7] Syrjanen, R. K., Kilpi, T. M., Kaijalainen, T. H., Herva, E. E. & Takala, A. K. Nasopharyngeal carriage of *Streptococcus pneumoniae* in Finnish children younger than 2 years old. *J. Infect. Dis.* **184**, 451–459 (2001). arXiv:http://jid.oxfordjournals.org/content/184/4/451.full.pdf+html.

[8] Bretó, C., He, D., Ionides, E. L. & King, A. A. Time series analysis via mechanistic models. *Ann. Appl. Stat.* **3**, 319–348 (2009).

[9] He, D., Ionides, E. L. & King, A. A. Plug-and-play inference for disease dynamics: measles in large and small populations as a case study. *J. Roy. Soc. Interface* **7**, 271–283 (2010).

[10] King, A. A. *et al.* pomp: Statistical inference for partially observed markov processes (R package). http://pomp.r.forge.r-project.org (2010).

[11] Ionides, E. L., Bretó, C. & King, A. A. Inference for nonlinear dynamical systems. *Proc. Natl. Acad. Sci. U. S. A.* **103**, 18438–18443 (2006).

[12] Ionides, E. L., Breto, C. & King, A. A. *Modeling Disease Dynamics: Cholera as a Case Study*, chap. 8, 13—140 (Wiley, Hoboken NJ, 2007).

[13] King, A. A., Ionides, E. L., Pascual, M. & Bouma, M. J. Inapparent infections and cholera dynamics. *Nature* **454**, 877–881 (2008).

[14] Arulampalam, M. S., Maskell, S., Gordon, N. & Clapp, T. A tutorial on particle filters for online nonlinear/non-gaussian bayesian tracking. *IEEE transactions on signal processing* **50**, 174—188 (2002).

[15] Bogaert, D., De Groot, R. & Hermans, P. *Streptococcus pneumoniae* colonisation: the key to pneumococcal disease. *Lancet Infect. Dis.* **4**, 144–154 (2004).

[16] Ferreira, D., Jambo, K. & Gordon, S. Experimental human pneumococcal carriage models for vaccine research. *Trends Microbiol.* **19**, 464–470 (2011).

[17] Gray, B., Converse, G. & Dillon, H. Epidemiologic studies of *Streptococcus pneumoniae* in infants: acquisition, carriage, and infection during the first 24 months of life. *J. Infect. Dis.* **142**, 923–933 (1980).

[18] Weinberger, D. M. *et al.* Association of serotype with risk of death due to pneumococcal pneumonia: A meta-analysis. *Clin. Infect. Dis.* **51**, 692–699 (2010).
